# Multiscale protein networks systematically identify aberrant protein interactions and oncogenic regulators in seven cancer types

**DOI:** 10.1186/s13045-023-01517-2

**Published:** 2023-12-15

**Authors:** Won-Min Song, Abdulkadir Elmas, Richard Farias, Peng Xu, Xianxiao Zhou, Benjamin Hopkins, Kuan-lin Huang, Bin Zhang

**Affiliations:** 1 Department of Genetics and Genomic Sciences, Icahn School of Medicine at Mount Sinai, One Gustave L. Levy Place, 1425 Madison Avenue, New York, NY 10029 USA; 2 Mount Sinai Center for Transformative Disease Modeling, Icahn School of Medicine at Mount Sinai, One Gustave L. Levy Place, New York, NY 10029 USA; 3 Icahn Genomics Institute, Icahn School of Medicine at Mount Sinai, One Gustave L. Levy Place, New York, NY 10029 USA; 4 Department of Physiology and Biophysics, The Englander Institute for Precision Medicine, Weill Cornel Medicine, New York, NY 10065 USA; 5 Department of Pharmacological Sciences, Icahn School of Medicine at Mount Sinai, One Gustave L. Levy Place, New York, NY 10029 USA

## Abstract

Global proteomic data generated by advanced mass spectrometry (MS) technologies can help bridge the gap between genome/transcriptome and functions and hold great potential in elucidating unbiased functional models of pro-tumorigenic pathways. To this end, we collected the high-throughput, whole-genome MS data and conducted integrative proteomic network analyses of 687 cases across 7 cancer types including breast carcinoma (115 tumor samples; 10,438 genes), clear cell renal carcinoma (100 tumor samples; 9,910 genes), colorectal cancer (91 tumor samples; 7,362 genes), hepatocellular carcinoma (101 tumor samples; 6,478 genes), lung adenocarcinoma (104 tumor samples; 10,967 genes), stomach adenocarcinoma (80 tumor samples; 9,268 genes), and uterine corpus endometrial carcinoma UCEC (96 tumor samples; 10,768 genes). Through the protein co-expression network analysis, we identified co-expressed protein modules enriched for differentially expressed proteins in tumor as disease-associated pathways. Comparison with the respective transcriptome network models revealed proteome-specific cancer subnetworks associated with heme metabolism, DNA repair, spliceosome, oxidative phosphorylation and several oncogenic signaling pathways. Cross-cancer comparison identified highly preserved protein modules showing robust pan-cancer interactions and identified endoplasmic reticulum-associated degradation (ERAD) and N-acetyltransferase activity as the central functional axes. We further utilized these network models to predict pan-cancer protein regulators of disease-associated pathways. The top predicted pan-cancer regulators including RSL1D1, DDX21 and SMC2, were experimentally validated in lung, colon, breast cancer and fetal kidney cells. In summary, this study has developed interpretable network models of cancer proteomes, showcasing their potential in unveiling novel oncogenic regulators, elucidating underlying mechanisms, and identifying new therapeutic targets.

**Supplementary Information:**

The online version contains supplementary material available at 10.1186/s13045-023-01517-2.

To the Editor

Dysregulated proteins play a critical role in the development of tumors, but many large-scale -omics studies predominantly centered around transcriptomics which has some substantial discordance with proteomics [1–3]. Hence, systematic identification of proto-oncogenic proteins is crucial. Herein, we developed multiscale protein co-expression networks from a large cohort of proteomic datasets in seven cancers [4] including breast carcinoma (BRCA), clear renal cell carcinoma (CCRCC), colorectal carcinoma (CRC) [5], hepatocellular carcinoma (HCC) [6], lung adenocarcinoma (LUAD), stomach cancer (STAD) [7], and uterine corpus endometrial carcinoma (UCEC) (Fig. 1A, D; Table S1) to dissect the proteomic landscape of oncogenic pathways (Additional file 1: Fig. S1).

**Fig. 1:**
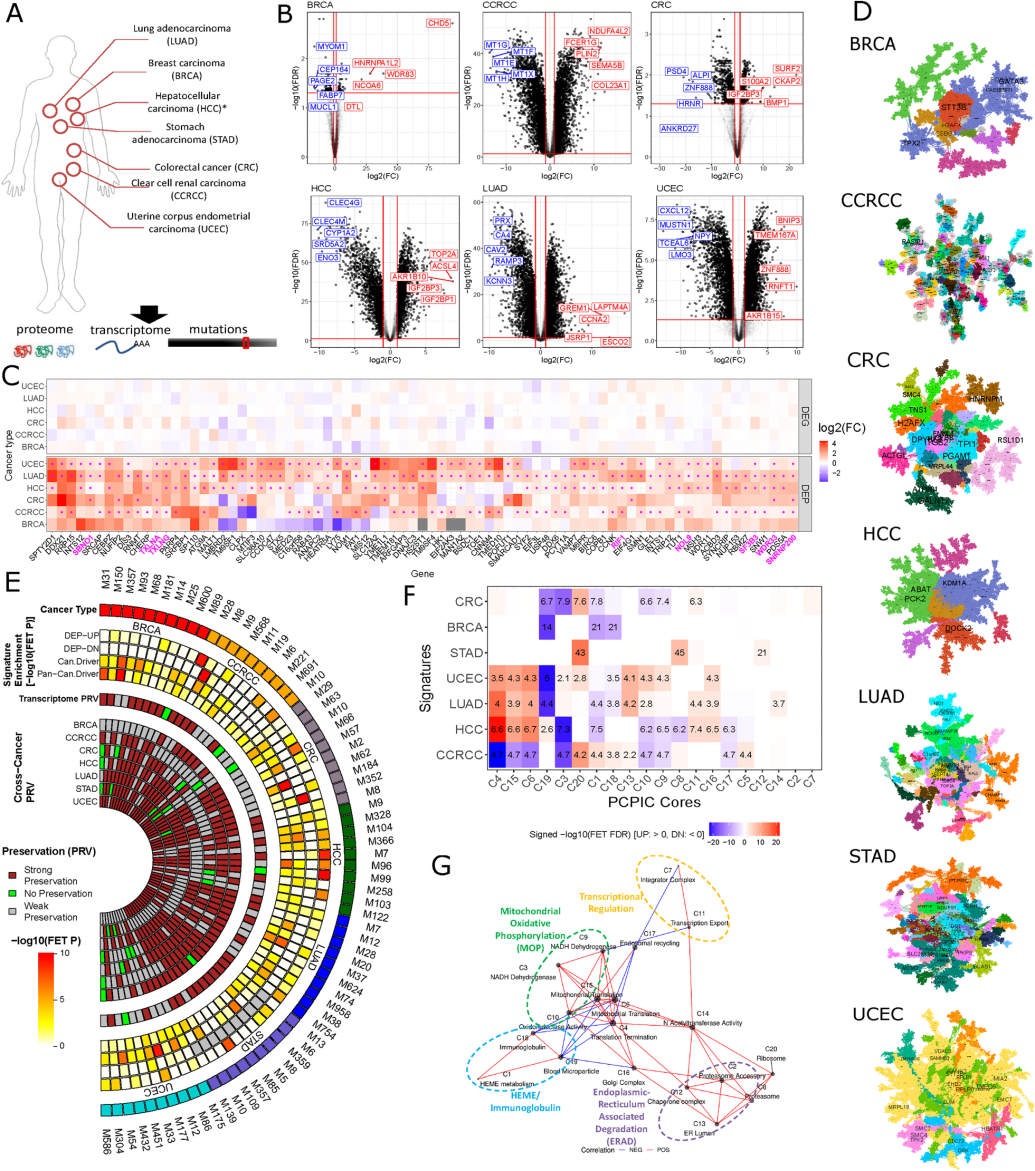
Integrative network analysis of pan-cancer protein interactomes. **A** Data curation. The diagram illustrates omics data types (proteome, transcriptome and mutation) in seven cancer types analyzed in this study, and **B** Volcano plots of DEPs in tumors. The top 5 up- or down-regulated DEPs in each cancer type are labeled. **C**. Proteome-specific DEPs: Differential expressions of DEPs in the respective cancer transcriptomes were compared to derive proteome-specific DEPs. The most recurrent proteome-specific DEPs in at least three cancer types were identified by Super Exact Test [11] (Fig. S3D), and they are highlighted in magenta color. **D** Global protein co-expression networks of seven cancer types. The top network hubs are highlighted and the modules at the resolution of α = 1 are shown as different colored nodes. **E** Molecular characteristics of the top 10 protein modules in each cancer type. The tracks from the outer most one to the inner most one represent module names (1), cancer type (2), enrichment of the DEP signatures in each cancer type (3, 4), enrichment of the mutational drivers in each cancer type (5), enrichment of the pan-cancer mutational drivers (6), preservation of the protein modules in the respective transcriptomics data (Transcriptome PRV; 7), and preservation of protein modules in the proteomics data of the other cancer types (Cross-cancer PRV; 9–15). There are three scenarios for module preservation: “strong preservation” represented by brown block, “no preservation” by a green block, and “weak preservation” by a grey block. The color intensity bar on the left of the circus plot represents –log10(Fisher’s Exact Test p-value). **F** Enrichment of the DEP signatures in pan-cancer protein interactomes represented by Pan-cancer protein interaction communites (PCPICs). **G** Cross-talk among the pan-cancer protein interactomes. In the network, each node represents a PCPIC core and the red and blue links denote positive and negative correlations, respectively. The most enriched pathway for each PCPIC is provided

Using the matched adjacent normal samples of the same organs from the Clinical Proteomic Tumor Analysis Consortium (CPTAC), we first identified differentially expressed proteins (DEP) in all the cancer types except STAD for which there are no matched adjacent normal samples (Fig. 1B; Additional file 1: Table S2). The DEP signatures were enriched for several hallmark pathways including up-regulation of cell cycle-associated (G2M checkpoints, E2F targets) and oncogenic MYC/MTORC1 signaling pathways, and down-regulation of myogenesis, adipogenesis, coagulation and heme metabolism pathways (Additional file 1: Fig. S2A). The up-regulated DEP signatures were also enriched for the essential genes identified from CRISPRi screening in the respective cancer cell lines [8] (Additional file 1: Fig. S3A). Compared to the respective transcriptomics, some DEPs were proteome-specific across multiple cancer types (Additional file 1: Table S3) and these proteins were involved in epigenetic and post-transcriptional regulations (Fig. 1C) including chromatin modification (SBNO1), intracellular vesicle trafficking (TXLNA, TXLNG), DNA repair (RIF1), post-transcriptional regulations including RNA editing (ADAR), RNA binding (NUFIP2), pre-mRNA 3′ end processing (WDR33), spliceosome (SNRNP200, SF3B3) and rRNA processing (NOL9). In LUAD, the expressions of the proteome specific DEPs showed distinctive prognostic associations in comparison to the respective transcriptome (Additional file 1: Supplemental Results; Fig. S4).

Through the protein co-expression network analysis (Additional file 1: Table S4), we identified the co-expressed protein modules enriched for the known mutational drivers from the Pan-cancer atlas study [9] and the DEP signatures for each cancer type except STAD (Fig. 1E). The hub proteins in the top oncogenic modules included several known mutational drivers such as GATA3 in breast cancer, CDH1 and CTNND1 in UCEC (Additional file 1: Supplemental Results; Fig. S5). Several proteome-specific modules were differentially expressed in tumors and they were involved in KRAS-driven HEME metabolism (Additional file 1: Fig. S6C), spliceosome interacting with mutational drivers in chromatic remodeling (Additional file 1: Fig. S6D), DNA single-strand break repair (Additional file 1: Fig. S6E), and FAT1-driven mitochondrion (Additional file 1: Fig. S6F).

Comparison of the seven protein co-expression networks identified 20 modules preserved across the seven cancer types (Additional file 1: Supplemental Results; Table S5). These conserved modules, termed as pan-cancer protein interaction communities (PCPIC) (Additional file 1: Methods; Fig. S7), represent the essential functional components of commonly co-expressed proteins (Additional file 1: Fig. S8; Table S5). The PCPIC cores showed distinct differential protein expression patterns, dependent on cancer types (Fig. 1F; Additional file 1: Supplemental Results), and constituted a PCPIC network (Fig. 1G). The PCPIC network harbors a number of key pathways such as mitochondrial oxidative phosphorylation (MOP), endoplasmic reticulum-associated degradation (ERAD), transcriptional regulation, and HEME/immunoglobulin. The ERAD and MOP axes were bridged by post-translational mechanisms such as golgi complex and N-acetyltransferase pathways (Fig. 1G).

We identified potential oncogenic regulators as highly connected proteins with the dys-regulated pathways [10], i.e. the DEP signatures (Additional file 1: Methods). The top pan-cancer regulators (Fig. 2A) included DDX21 interacting with RNA binding proteins in rRNA processing and transcriptions (Fig. 2B), RSL1D1 interacting with oncogenic MYC-regulated pathways in multiple cancers (Fig. 2C), and SMC2 interacting with cell cycle pathways and EZH2-modulated epigenetic regulations (Fig. 2D).

**Fig. 2:**
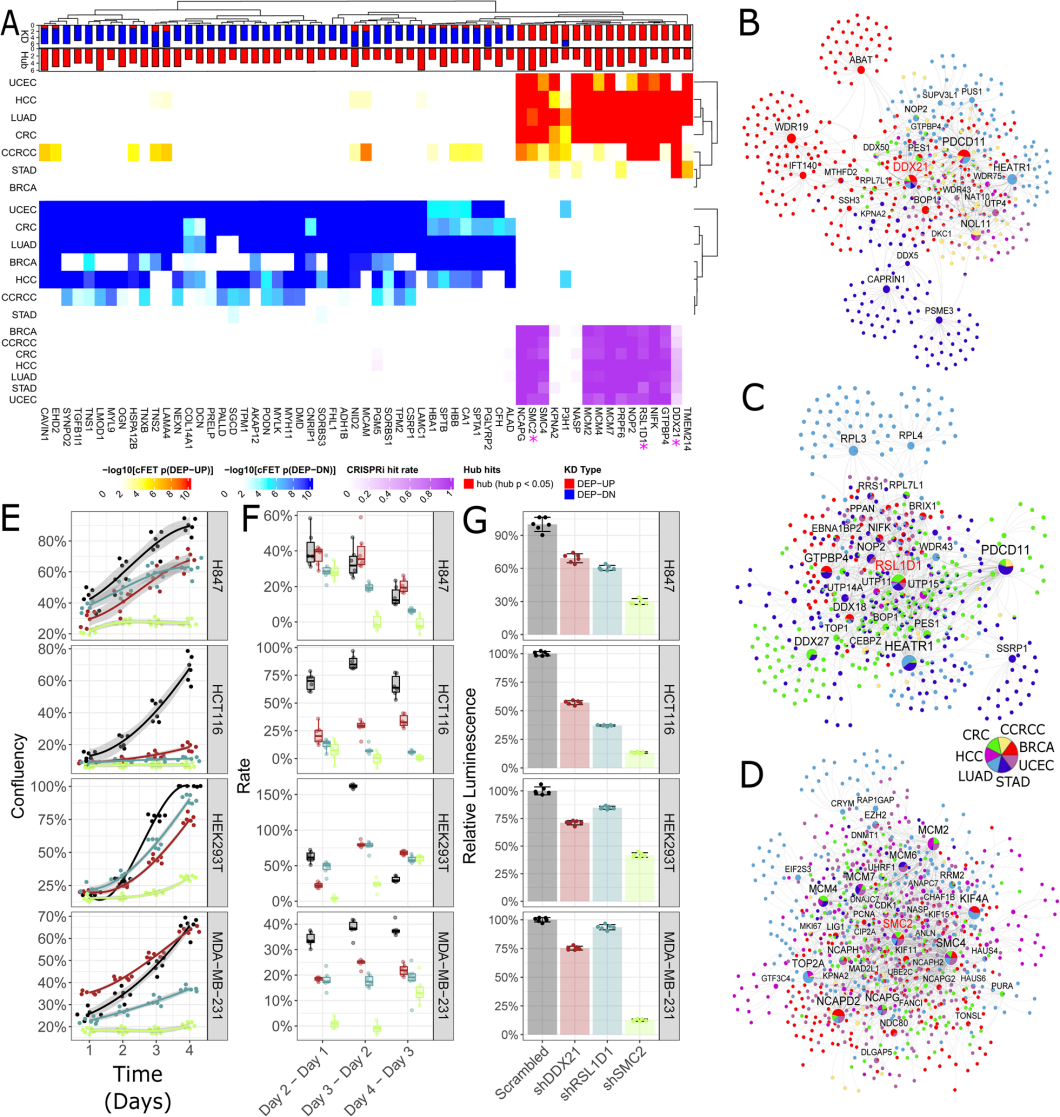
Identification of pan-cancer proteomic regulators. **A** The top pan-cancer protein network drivers. The top bar shows the frequencies of up- and down-regulations of each pan-cancer protein driver in the seven cancers while the 2nd bar from the top shows the frequency of the hub status of each protein driver in the seven cancers. The first and second heat maps from the top represent the enrichment of the up- and down-regulated cancer-type-wise DEP signatures in the neighborhoods of the protein drivers, respectively. The color intensity is proportional to –log10(FDR corrected FET *p* value). The bottom heatmap summarizes the percentage of significant hits for each protein driver in the CRISPRi screening of cancer cell lines from Archilles database with FDR < 0.05. **B**–**D** Pan-cancer neighborhood networks of the top-ranked novel regulators, DDX21 (**B**), RSL1D1 (**C**), and SMC2 (**D**). The links are color-coded by the cancer types. The piechart of each node shows the proportions of links from different cancer types. **E**–**G** Anti-tumor activities by silencing the predicted pan-cancer proteome regulators, RSL1D1, DDX21 and SMC2. We conducted shRNA knock-down of the predicted regulators in lung cancer (H847), colon cancer (HCT116), fetal kidney (HEK293T) and breast cancer (MDA-MB-231), with the scrambled shRNAs as controls. **E** Confluence of different cancer cells transfected by shRSL1D1 (light blue), shDDX21 (brown) and shSMC2 (green), compared to the scrambled control (Scrambled, black). The confluences (y-axis) were measured from day 1 to day 4 (x-axis). **F** Rate of confluence change in subsequent days. Cases showing significantly lower rate of change, compared to the scrambled control, are marked by red asterisks with different levels of significance shown at the top legend. **G** Relative cell viability change in comparison to the scrambled control by CTG luminescent cell viability assay

shRNA knockdowns of several top key protein regulators in cancer cell lines including H847 (lung), HCT116 (colon), MDA-MB-231 (breast cancer), and HEK293T (fetal kidney) significantly reduced cell growth (Fig. 2E; Additional file 1: Experimental Procedure and Method) except shDDX21 in MDA-MB-231 due to the poor knock-down efficiency (86.3%). The growth rates and cell viability temporally slowed down in all the four cell lines (Fig. 2F,G). Overall, silencing the pan-cancer oncogenic regulators induced significant anti-tumor activities across multiple cancer types, validating some key predictions from our pan-cancer protein network analysis.

In summary, the pan-cancer proteomic network models developed in this study can serve as a blueprint for further investigation into the oncogenic mechanisms.

### Supplementary Information

**Additional file 1. Supplemental Results; Methods; Supplemental Figure Legend; Fig. S1.** The overall workflow of pan-cancer proteome network analysis; **Fig. S2.** Enriched hallmark pathways in DEP signatures; **Fig. S3.**. Overview of differentially expressed proteins; **Fig. S4.**. Predictive power of mRNA and protein expressions of proteome-specific DEPs; **Fig. S5.**. Subnetworks of the top protein modules in each cancer type in Fig. 1E; **Fig. S6.**. Preserved or proteome-specific co-expressed protein modules in the respective transcriptome; **Fig. S7.**. Workflow of the Pan-cancer protein interaction community (PCPIC) analysis; **Fig. S8.**. Most enriched pathways in PCPIC cores; **Fig. S9.**. Cross-talk across distinct PCPICs; **Fig. S10.**. Top hub genes in protein co-expression networks; **Fig. S11.**. Enrichment of various protein signatures in the cancer essential genes identified by the in vitro screening in the Archilles database; **Fig. S12.**. Comparison of proteome (PR) and transcriptome (TX) network connectivity in each cancer type; **Fig. S13.**. Enriched hallmark pathways in proteome- (PR) or transcriptome-(TX) specific hub genes, or shared hub genes in PR and TX; **Fig. S14.**. Validated drivers by gene perturbations signatures in cancer cells from LINCS database; **Fig. S15.**. Evaluation of TCGA Pan-cancer atlas (PanCanAtlas) and CPTAC transcriptome (TX) cohorts for proteome module preservation analysis; **Supplemental Table Legend; Table S1**. Description of the cancer proteome datasets; **Table S2**. Summary of differentially expressed protein (DEP) signatures; **Table S3**. The number of differentially expressed proteins (DEPs) in each cancer type and the number of proteome specific DEPs, i.e., DEPs without differential expression at the mRNA level in the respective tumor transcriptome; **Table S4**. Numbers of protein and mRNA modules by MEGENA; **Table S5**. List of core proteins in Pan-cancer protein interaction communities (PCPICs)

## Data Availability

CPTAC proteogenomic cohorts data are available via CPTAC data portal: https://cptac-data-portal.georgetown.edu/cptacPublic/. The RNA-seq data from the Pan-cancer Atlas consortium are available via: https://gdc.cancer.gov/about-data/publications/pancanatlas. In addition, the RNA-seq data for CPTAC cohorts are available at the GDC data portal: https://portal.gdc.cancer.gov/. The differentially expressed proteins, protein co-expression networks and regulator prediction data are available at: https://doi.org/10.5281/zenodo.8231217. The codes and processed data to reproduce the figures are available at: https://zenodo.org/badge/latestdoi/491634149.

## Abbreviations

MS: Mass spectrometry
BRCA: Breast cancer
CRC: Colorectal cancer
UCEC: Uterine corpus endometrial carcinoma
MB: Medulloblastoma
CCRCC: Clear cell renal carcinoma
HCC: Hepatocellular carcinoma
HBV: Hepatitis B virus
HBV-HCC: HBV-infected hepatocellular carcinoma
LUAD: Lung adenocarcinoma
STAD: Stomach cancer
rRNA: Ribosomal RNA
CRISPRi: CRISPR interference
PCPIC: Pan-cancer protein interaction community
DEP: Differentially expressed protein
DEG: Differentially expressed gene
FET: Fisher’s exact test
FDR: False discovery rate
PRV: Preservation
