# Supplementary material for: Multiscale protein networks systematically identify aberrant protein interactions and oncogenic regulators in seven cancer types
Source: J Hematol Oncol. 2023 Dec 15;16:120. doi: 10.1186/s13045-023-01517-2 (PMC10724946; doi:10.1186/s13045-023-01517-2)
Supplement: Supplementary file 1 — Additional file 1. Supplemental Results; Methods; Supplemental Figure Legend; Fig. S1. The overall workflow of pan-cancer proteome network analysis; Fig. S2. Enriched hallmark pathways in DEP signatures; Fig. S3.. Overview of differentially expressed proteins; Fig. S4.. Predictive power of mRNA and protein expressions of proteome-specific DEPs; Fig. S5.. Subnetworks of the top protein modules in each cancer type in Fig. 1E; Fig. S6.. Preserved or proteome-specific co-expressed protein modules in the respective transcriptome; Fig. S7.. Workflow of the Pan-cancer protein interaction community (PCPIC) analysis; Fig. S8.. Most enriched pathways in PCPIC cores; Fig. S9.. Cross-talk across distinct PCPICs; Fig. S10.. Top hub genes in protein co-expression networks; Fig. S11.. Enrichment of various protein signatures in the cancer essential genes identified by the in vitro screening in the Archilles database; Fig. S12.. Comparison of proteome (PR) and transcriptome (TX) network connectivity in each cancer type; Fig. S13.. Enriched hallmark pathways in proteome- (PR) or transcriptome-(TX) specific hub genes, or shared hub genes in PR and TX; Fig. S14.. Validated drivers by gene perturbations signatures in cancer cells from LINCS database; Fig. S15.. Evaluation of TCGA Pan-cancer atlas (PanCanAtlas) and CPTAC transcriptome (TX) cohorts for proteome module preservation analysis; Supplemental Table Legend; Table S1. Description of the cancer proteome datasets; Table S2. Summary of differentially expressed protein (DEP) signatures; Table S3. The number of differentially expressed proteins (DEPs) in each cancer type and the number of proteome specific DEPs, i.e., DEPs without differential expression at the mRNA level in the respective tumor transcriptome; Table S4. Numbers of protein and mRNA modules by MEGENA; Table S5. List of core proteins in Pan-cancer protein interaction communities (PCPICs) [file 13045_2023_1517_MOESM1_ESM.docx]

**SUPPLEMENTAL RESULTS**

***Overall Study Design***

We developed a network biology centered framework to analyze the pan cancer proteomics data comprised of 687 cases across 7 cancer types including BRCA (115 tumor samples; 10,438 genes), CCRCC (100 tumor samples; 9,910 genes), CRC (91 tumor samples; 7,362 genes), HCC (101 tumor samples; 6,478 genes), LUAD (104 tumor samples; 10,967 genes), STAD (80 tumor samples; 9,268 genes), and UCEC (96 tumor samples; 10,768 genes) with matched normal samples where available (**Fig. S1A**; **Table S1**).

Firstly, we identified protein signatures of tumor proteomes to guide the search for aberrantly altered subnetworks (**Fig. S1B**). Except for STAD, we leveraged on the availability of the matched normal samples to systematically survey altered pathways in tumor as differentially expressed proteins (DEPs). For STAD, the samples lacked matched normal samples, and we resorted to DEPs between Epstein Barr virus (EBV) + and EBV- samples as a proxy for distinctive tumor signatures^1^. These DEPs were subsequently compared across different cancer proteomes (cross-cancer DEPs) and against the respective cancer transcriptome (proteom-specific DEPs).

Then, we constructed integrative cancer protein network models and catalogued shared protein interactomes across cancers or respective cancer transcriptome, and dys-regulated subnetworks in data-driven manner (**Fig. S1C**). For each cancer proteome, we performed quality controls to remove outliers and adjust confounding variables in the proteome data (see *Data Quality Control for Network Analysis* in the **Materials and Methods**), then applied Multi-scale Gene co-Expression Network Analysis (MEGENA)^2^ to identify the protein co-expression networks. These *de novo* protein interaction networks were compared across cancer types to identify highly conserved protein module via Pan-cancer Protein Interaction Community (PCPIC) analysis (see **Materials and Methods**). In tandem, for each cancer type, we constructed the respective transcriptome co-expression network models to identify protein modules present in the transcriptome, or unique to the cancer proteome (see **Materials and Methods**). The dys-regulated protein subnetworks were also identified by evaluating the enrichments of the DEP signatures and mutational driver signatures (curated from Pan-cancer Atlas study by TCGA^2^). The dys-regulated subnetworks were determined with FDR-adjusted Fisher’s Exact Test (FET) p-value < 0.05 in the co-expressed protein modules.

The protein network models were then jointly utilized to identify the pan-cancer pro-tumorigenic regulators, and validated *in vitro* (**Fig. S1D**). Assuming the protein networks capture the protein-protein interactions, we hypothesized the central proteins interacting in proximity with the dys-regulated pathways captured in the DEP signatures would play central roles. Thus, we investigated the protein network topology in search of proteins showing high connectivity with the DEP signatures in different cancer protein networks via Key Driver Analysis (see **Materials and Methods**). The top 3 genes were systematically prioritized and validated *in vitro* across different cancer cell lines, to evaluate the ubiquitous (proto-)oncogenic roles in pan-cancer context.

***Prognostic significance of proteome-specific differentially expressed proteins***

It’s been reported that differentially expressed proteins in tumors are more often associated with patient survival than the respective RNA expression in lung adenocarcinoma^3^. To systematically evaluate this, we prioritized 79 proteome-specific differentially-expressed proteins (DEPs) from the LUAD cohort and evaluated their predictive power for patients' survival. The LUAD cohort included 12-month follow-up for 98 patients with protein expressions (Dead: 10; Alive: 88) and 99 patients with mRNA expressions (Dead: 8; Alive: 89). With the comparable numbers of clinical follow-up samples and observed deaths at 12 months, the cohort served as an ideal data set to comparatively evaluate the prognostic significance of the proteome-specific DEPs with the respective mRNA expressions.

We employed logistic regression to assess the predictive power of the proteome-specific DEPs for patient survival. The model included patient survival (being lost at one-year follow-up) as the dependent variable and protein expression levels as the primary independent variable, adjusting for age, gender, race, and ethnicity as covariates. A similar approach was used for transcriptomic data, with mRNA levels as the primary independent variable. The significance of each protein and mRNA’s association with patient survival was determined using z-values and corresponding p-values. The false discovery rate (FDR) was also calculated to account for multiple testing (Benjamini-Hochberg). The regression coefficients (Estimates) provided a measure of the change in the log odds of observed death in 12-month follow-up (i.e. death risk in 12 months) for a one-unit increase in protein or mRNA expression. A negative coefficient indicated a decrease in the death risk with increased expression, while a positive coefficient indicated an increase in these odds. Comparing the survival associations of the protein expressions and the respective mRNA expressions among the proteome-specific DEPs, we observed that their protein expressions were significantly more down-regulated in the patients with observed deaths within 12-months, than the respective mRNA expressions (**Fig. S4**A**;** Paired T-test p-value = 3.71E-2).

Some of the protein expressions showed prognostic significance in proteome-specific manner. While mRNA expressions of SYF2 did not show significant differential expressions between dead and alive patients at the 12-month follow-up (**Fig. S4B;** T-test p-value = 1.57E-1), the protein expressions of SYF2 were associated to poor outcome (**Fig. S4C;** T-test p-value = 1.60E-2). Conversely, protein expressions of MAN1A2 were associated to good outcomes (**Fig. S4E**; T-test p-value = 7.07E-3), its mRNA expressions were not significantly associated to the 12-months survival (**Fig. S4D**; T-test p-value = 7.14E-1).

Overall, these data show protein expressions of the proteome-specific DEPs present distinctively different biomarkers to predict patients’ outcomes, compared to the respective transcriptome.

***Protein co-expression networks: Data-driven molecular models of functional pathways in tumors***

We constructed the co-expression protein networks for the seven cancer types, using Multi-scale Gene co-Expression Network Analysis (MEGENA)^3^ (**Fig. 1D**). After quality controls to remove outliers and adjust confounding variables in the proteome data (see *Data Quality Control for Network Analysis* in the **Methods**), MEGENA was performed for each cancer type proteome to yield sparse co-expression network with a hierarchy of parent and child co-expressed modules with varying compactness, and significant hub proteins^2^. Similarly, we constructed gene co-expression networks from respective transcriptome data from TCGA. The constructed protein networks are shown in **Fig. 1D**, and are summarized with the transcriptome networks in **Table 2** (see *Protein/Gene Co-expression Network Analysis* in **Methods**).

Then, we interrogated network connectivity of individual proteins, and co-expressed protein modules to catalogue **shared pathways** **across cancer types** (i.e. pan-cancer pathways, **Fig. S1C**-ii), **proteome-specific pathways, compared to transcriptome** (**Fig. S1C**-iii), **disease-associated pathways** by enrichment of differentially expressed proteins (DEPs) in tumor, then predicted **pan-cancer** **key regulators** as up-stream regulators of disease-associated pathways in the network models (**Fig. S1D**). We experimentally validated pan-cancer regulators by loss-of-function to confer anti-tumor effects in diverse cancer types (**Fig. S1D**): lung (H847), colon (HCT116) and breast (MDA-MB-231) cancer cells, in addition to fetal kidney cells (HEK293T).

***Systematic characterization of dys-regulated pathways in seven cancer proteomes***

Per cancer type, we systematically evaluated most aberrantly altered subnetworks in each cancer type. Briefly, we evaluated enrichments of DEP signatures and cancer type-specific/pan-cancer mutational driver signatures in the co-expressed protein modules. Then, the protein modules were prioritized by the overall enrichments of the signatures (see *Module Prioritization* in **Methods**). Summaries for enrichment features of the top 10 modules across the seven cancer types are shown in **Fig. 1E**.

A majority of the top 10 protein modules showed significant interactions in the respective transcriptome while few protein modules were unique to proteome (track 7; **Fig. 1E**). Also, many of these protein modules were shared across multiple cancer types while some modules were cancer type-specific (tracks 7-14; **Fig. 1E**).

The top enriched modules reflected some of key pro-tumorigenic pathways exploited in the seven cancer types (**Fig. S5**). Interestingly, these modules were commonly enriched for pan-cancer mutational drivers (labeled in magenta), and some of them coincided with the high connectivity proteins (i.e. hubs) in the subnetworks, suggesting them as potential (proto-)oncogenic drivers. For example, GATA3 is a pan-cancer mutational driver and a hub in breast cancer module, M31. It is a known transcriptional factor to drive ERα pathways in breast cancer, and is frequently mutated gene that drive breast cancer development^4^. In addition, PTMA (prothymosin α; driver in CRC), DMD (dystrophin; driver in STAD), CDH1 and CTNND1 (drivers in cell junction module in UCEC) are other mutational driver hub proteins in other cancer types.

Several DEPs were also top hub proteins, emerging as the potential key regulators of the subnetworks. In CCRCC, RASIP1, a critical regulator of vascular-specific GTPase signaling and EMT in renal carcinoma^5^, is the most connected, up-regulated protein in the EMT module. In HCC, a collagen fiber assembly protein (BGN) is an up-regulated hub, and closely interacts with down-regulated cell adhesion/actin binding proteins (EMILIN1 - elastin microfibril interfacer 1, SYNPO2 - synaptopodin 2, COL14A1 – an adhesive collagen, CALD1 – actin and myosin binding protein). In LUAD, chromosome aggregation and segregation proteins such as condensin complex subunits (NCAPD2, SMC2), minichromosome maintenance complex components (MCM2-6) and kinesins (KIF4A, KIF11) were up-regulated hub proteins, and closely interacted with several down-regulated hub DEPs involved in diverse functions. These include nuclear protein import adaptor protein (KPNA2), mitochondrial damage repair regulator (SPATA18), myosin light chain kinase (MYLK, primarily expressed in actin filaments). In UCEC, a RAS oncogene (RAB1D) is the most connected hub and an up-regulated DEP and closely interacts with other up-regulated DEPs in cell-cell adhesion pathways such as ADAM9^6^ and mutational drivers (CDH1, CTNND1).

Overall, the co-expressed protein modules reflect key pro-tumorigenic pathways in the seven cancers, and shed lights on the crucial regulators as highly connected hub proteins.

***Protein co-expression networks reveal proteome-specific pathways in tumor***

Our current knowledge on discordant mRNA-protein pairs are largely dependent on known pathways in the literature. Some studies remarked highly concordant mRNA-protein pairs were mostly enriched in pathways such as secreted proteins and time-dependent cell cycles requiring fast translations to execute their functions^7^, or metabolic genes that show high stability^8^. On the other hand, proteins involved in chromatic modification and transcriptional regulations rapidly degrade, and showed discordance with the more stable mRNA counterparts^2^.

In this study, we surveyed for proteome-specific modules by an unbiased, data-driven approach. Specifically, for each proteome module, we performed module preservation analysis to test significant gene-gene correlations in the respective transcriptome (see *Module Preservation Analysis* in the **Methods**). The protein modules whose interactions were absent in the transcriptome were identified as “proteome-specific” modules for further analysis.

Overall, the transcriptomic preservation of the protein modules showed multi-scale patterns. Although some large parent modules were transcriptomically preserved, several child modules were not transcriptomically preserved, hence were proteome-specific modules (**Fig. S6A**).

Among them, several proteome-specific modules were up-regulated in tumors, and associated with known functions and pathways (**Fig. S6**). They include M666 in CCRCC (heme metabolism; **Fig. S6C**), M99 in HCC (spliceosome; **Fig. S6D**), M191 in LUAD (DNA single-strand break repair; **Fig. S6E**) and M428 in UCEC (constituent parts of mitochondrion; **Fig. S6F**). Some of these modules harbored mutational drivers and were associated with known oncogenic pathways.

The heme metabolism module, M666, in CCRCC included a mutation driver gene, KRAS (**Fig. S6C**). Accordingly, KRAS oncogenic signaling was present in the proximity of M666 within the CCRCC protein network. The up-regulated genes in KRAS mutant epithelial kidney cancer cell lines were enriched in M8, the M666’s parent module (Bonferroni adjusted FET p=1.55E-2, 2.73 FE). Further, M8 was enriched for NADH dehydrogenase complex, suggesting exploitation of mitochondrial respiratory chain for the concomitant energy production by tumor cells.

The spliceosome module, M99, in HCC included several driver mutations involved in chromatin remodeling (KMT2D, SMAD2, SMAD4), TSC complex (TSC1, TSC2) and programmed cell death (CASP2). The parent module of M99 in HCC, M10, was enriched for cell cycle pathways including E2F targets (Bonferroni adjusted FET p=4.28E-27, 2.82 FE) and G2M checkpoint (Bonferroni adjusted FET p=2.22E-13, 2.41 FE). Thus, the network model suggests multiple driver mutations synergize to activate aberrant spliceosomes in HCC, leading to proliferative advantages to tumors in HCC.

The mitochondrion module, M428 in UCEC, included frequently mutated driver, FAT1, whose loss-of-function may induce aberrant endometrial proliferation. Indeed, FAT1 acts as molecular brake on mitochondrial respiration, regulating vascular smooth muscle cell (SMC) proliferation in response to vascular damage, and FAT1 loss-of-function caused increased oxygen consumption and SMC proliferation^9^.

To sum up, the protein network models systematically identified novel proteome-specific pathways regulated by mutational drivers, and reveal new therapeutic avenues that are otherwise missed in transcriptome studies.

***Pan-cancer proteome modules: commonly exploited pathways across cancers***

While cancers are known to possess shared hallmark features across different cancer types^10-12^, these hallmark pathways were derived primarily from the findings in the literatures and may overlook disease contexts. Herein, we utilized the co-expressed protein modules from the seven cancers to systematically derive cross-cancer preserved protein interactions. To this end, we developed pan-cancer protein interaction community (PCPIC) approach to identify these co-expressed protein modules (**Fig. S7**; see P*an-cancer protein interaction community (PCPIC): Conserved protein modules across multiple cancer types* in **Methods**). Briefly, we searched for overlapping co-expressed modules across different cancer types, forming a module of overlapping modules, termed PCPIC. Within each PCPIC, we identified a core group of proteins shared by different cancer types and they form the PCPIC core, yielding 20 PCPIC cores (**Table S5**).

The PCPIC cores reflect distinct and closely interacting proteins in multiple cancer proteomes. They were enriched for distinct pathways and functions curated in the MSigDB database (**Fig. S8**), and the members in each core were highly interconnected in the Protein-Protein Interactions (PPI) network curated by the STRING database (**Fig. S9A**)^13^.

The PCPIC cores were differentially expressed in several tumor proteomes (**Fig. 1F**). Mitochondrial translation cores (C4, 6 and 15) were up-regulated simultaneously in HCC, LUAD and UCEC, and down-regulated in CCRCC. HEME, immunoglobulin and post-transcriptional regulation (PTR) cores (C1, 14 and 18) were commonly down-regulated in BRCA, LUAD and HCC, and up-regulated in CCRCC. Mitochondrial oxidative phosphorylation cores (MOP; C9 and 10) were commonly down-regulated in CRC, HCC and CCRCC, and up-regulated in LUAD and UCEC. Transcription export (TREX) complex another HEME core (C11 and 19) were commonly up-regulated in HCC and LUAD. Lastly, ribosome core (C20) showed the most prevalent down-regulations in multiple cancers including CRC, BRCA, UCEC and LUAD.

Regarding the PCPIC cores as the proxy for pan-cancer pathways, they showed robust patterns of cross-talks in the seven cancer proteomes. Briefly, we calculated the overall up- or down-regulation of each PCPIC core in each sample’s proteome as the Gene Set Variation Analysis (GSVA) z-score^14^, then evaluated the correlations among the GSVA z-scores to evaluate the cross-talks among the cores (see P*an-cancer Protein Interaction Community (PCPIC) Analysis* in **Methods**). Then, we identified the significantly correlated core pairs in at least four cancer types as PCPIC interactions.

Overall, the PCPIC network captured key functional interacting pathways cross the seven cancers (**Fig. 1G**). Mitochondrial ribosome subunits (C4, C6 and C15) and NADH-linked mitochondrial electron transport chain (C3, C9 and C10) closely interacted and represented the MOP component. HEME pathways (C1 and 19) and immunoglobulin (C18) cores were closely interacting, and were negatively correlated with the mitochondrial oxidative phosphorylation (MOP) components. Another major component is endoplasmic reticulum-associated degradation (ERAD) including C2/C8 (proteasomes to degrade misfolded proteins), C12 (chaperonin-containing T-complex) and C13 (endoplasmic reticulum chaperone). Notably, golgi complex (C16) and N-acetyltransferase pathway (C14) served as the bridge between ERAD axis and MOP axis, HEME and immunoglobulin pathways. Given the post-translational regulatory roles of golgi apparatus^15^ and N-acetyltransferases (cytosolic and ribosome-associated NatA)^16^, PCPI suggests the central roles played by post-translational regulations to mediate crucial pathway cross-talks.

***Protein network models identify key protein regulators of dys-regulated pathways in tumors***

Highly connected hubs in the networks are often potential regulators of closely connected genes and pathways^10^. Towards this end, MEGENA systematically identified protein network hubs with significantly high connectivity (hub p-value < 0.05; **Fig. S10**). The network hubs were essential to cancer. Compared to up-regulated DEPs or mutational drivers alone, the intersection of up-regulated DEPs and the hubs showed higher prediction accuracy for cancer essential genes (**Fig. S11**).

Comparison of the hubs across the seven cancer types identified cancer type-specific hubs as well as hubs shared by multiple cancer types (pan-cancer hubs). By clustering the proteins by connectivity patterns across the seven cancers, two groups of pan-cancer hubs emerged (**Fig. S10**): PANCAN-I which contains hubs in several cancer types, and PANCAN-II, highly connected hubs in most of the seven cancers. Some of the pan-cancer hubs were captured in PANCAN-II cancer driver proteins. For instance, two pan-cancer mutational drivers, CHD4 (chromodomain helicase DNA binding protein 4) and SF3B1 (splicing factor 3b subunit 1), were part of PANCAN-II hubs (**Fig. S10**).

In each cancer type, many genes showed different connectivity patterns in proteome and transcriptome (**Fig. S12**). We defined them as proteome-specific (proteome hub p-value < 0.05 & transcriptome hub p-value > 0.2), transcriptome-specific (proteome hub p-value > 0.2 & transcriptome hub p-value < 0.05) or transcriptome-proteome shared hubs with transcriptome (proteome hub p-value < 0.05 & transcriptome hub p-value < 0.05). These different categories of hubs were also enriched for distinct pathways and functions (**Fig. S13**). Epithelial-mesenchymal transition (EMT) pathway was one most enriched pathway in the transcriptome-proteome shared hubs, while oxidative phosphorylation and adipogenesis were most enriched in proteome-specific hubs across the seven cancers.

***Identification of novel pan-cancer pro-tumorigenic regulators***

Among the nominated regulators, we searched novel pan-cancer regulators whose pro-tumorigenic roles have not been well-known in the literature (see *Key Driver Analysis (KDA) and Top Regulator Nominations* in the **Methods**). After surveying for known pro-tumorigenic roles in the literature, we identified the top three candidates for further studies: SMC2 (a central component of condensin complex, required for mitotic-like condense chromosomes), RSL1D1 (also known as cellular senescence-inhibited gene, CSIG) and *DDX21* (DExD-box helicase 21) (**Fig. 2B-D**). These genes were also cancer essential genes as identified by CRISPRi screening in cancer cell lines across the seven cancers and their loss-of-function led to significant decreases in growth.

The pan-cancer regulators interacted with several known oncogenic pathways. The DDX21-centered subnetworks (**Fig. 2B**) harbor RNA binding proteins involved in rRNA processing and transcriptions, including RNA helicases (DDX5, DDX50, SUPV3L1), ribosome biogenesis factors (WDR43, WDR75, PDCD11, HEATR1, NOL11, UTP4, PES1) and ribosomal assembly (NOP2). In LUAD, the subnetwork was enriched for MYC-regulated pathways (FET FDR=4.86E-6, 62.2 EFC), and suggests oncogenic MYC-ribosome biogenesis axis^17^.

Similarly, the RSL1D1-centered subnetwork (**Fig. 2C**) is also associated with rRNA processing pathways including the helicases (DDX18, DDX27), ribosome biogenesis factor and assembly (WDR43, NOP2, BRIX1, PES1, PDCD1, HEATR1, GTPBP4), pre-rRNA processing (EBNA1BP2) and ribosome proteins (RPL3, RPL4, RRS1, RPL7L1). In multiple cancer networks, RSL1D1 interacts with NIFK, a nucleolar protein interacting with the FHA domain of proliferating cancer cell marker, Ki-67. NIFK and Ki-67 interaction is known to promote cell invation^18^.

The SMC2-centered network (**Fig. 2D**) is associated with oncogenic cell cycle pathways including E2F targets, G2M checkpoints, and DNA repair pathways (e.g. - FANCI, UHRF1, LIG1, CHAF1B). This subnetwork captures close interactions among several known epigenetic regulators such as EZH2^16^ in BRCA, LUAD and UCEC, polycomb repressive complex 2 (PRC2, an epigenetic suppressor) and UHRF1-DNMT1 (DNA methylation maintenance)^19^.

In summary, the pan-cancer regulators, DDX21, RSL1D1 and SMC2, regulate several oncogenic pathways including rRNA processing and cell cycle across different cancer types.

***Validation of pan-cancer protein network regulators***

We performed loss-of-function studies of the nominated regulators in multiple types of cancer cells to examine their functional importance. Specifically, gene expressions were knocked down by transfecting shRNAs for *SMC2*, *RSL1D1* and *DDX21* in lung (H847), colon (HCT116) and breast (MDA-MB-231) cancer cells, and fetal kidney (HEK293T).

These shRNAs significantly reduced cell growth and viability. In most cases, we observed significantly slowed or stopped in some cases, the growth of the transfected cancer cells compared to the scrambled controls (**Fig. 2E**). The exception is shDDX21 in MDA-MB-231 breast cancer cells, which may be due to relatively poor knock-down efficiency compared to the parental cells (86.3%). The slowed growth was also apparent from significantly reduced growth rates (**Fig. 2F**). Using the CellTitre-Glo luminescence viability assay, we also observed significantly reduced cell viability by the shRNAs in all cases (**Fig. 2G**).

Per target, shSMC2 showed the strongest anti-tumor effects by stalling the growth rates close to 0%, and decreasing viability under 30% for all cancer cells. shRSL1D1 slowed growth rates from 10% (in H847) to 80% (in HEK293T), and reducing cell viability from 30% (in HCT116) to 90% (in MDA-MB-231). shDDX2 slowed growth rates from 20% (in MDA-MB-231) to 80% (in HEK293T), and reduced cell viability from 60% (in HCT116) to 75% (in MDA-MB-231).

In summary, the significant anti-tumor activities of the nominated pan-cancer regulator knock-down across multiple cancer types demonstrate the protein networks as potent models to pinpoint pro-tumorigenic regulators.

**Discussion**

To the best of our knowledge, this study is the very first effort to construct multi-scale protein network models across the multiple cancer proteomes, and systematically identify pro-tumorigenic pathways using differentially expressed proteins (DEPs) in seven primary tumor cohorts. To this end, we first performed in-depth differential protein expression analysis between tumors and matched normal samples. We revealed that the DEPs were associated with not only known hallmark cancer pathways but also novel pathways such as chromatin modification, vesicle trafficking, RNA editing and rRNA processing.

The integration of the DEPs and the multiscale protein networks provided rich molecular models to predict key regulators as well as pro-tumorigenic mechanisms. This approach is advantageous over using DEPs alone to mine known pathways and functions by enrichments, as the knowledge-bases are biased towards pre-existing studies and may not capture the complexity of oncogenic pathways in full. Also, the predicted key drivers are more likely to be essential genes than DEPs.

Comparison of these proteomic cohorts and the corresponding TCGA pan-cancer transcriptomic datasets revealed proteome-specific DEPs, co-expressed protein modules and potential key protein regulators. Interestingly, many proteome-specific modules harbor mutational drivers. One example is the KRAS-driven heme metabolism pathway module in CCRCC (**Fig. S6C**). Recently, oncogenic KRAS signaling was found to induce ferrous iron (Fe2+) accumulations in tumor cells and was subsequently utilized to develop tumor-selective, iron-activable MEK inhibitor in KRAS-driven tumors^20^. In HBV-infected HCC, the modules associated with pre-mRNA splicing machineries (EFTUD2, SNRNP200, PRPF6/8, DDX23) contained mutational drivers in TGF-β (SMAD2/3)^21^ and mTORC1(TSC1/2)^22^ signaling pathways (**Fig. S6D**), suggesting convergent impact of oncogenic drivers on hyper-activated splicing in tumor. Under the presence of active TGF-β signaling, SMAD3 is induced to form a complex with PCBP1, and inhibits spliceosome assembly to favor the mesenchymal isoform of a cancer stem cell marker, CD44^21^. In UCEC, FAT1, a mutational driver and a molecular break for the mitochondrial respiratory chain pathway^9^, belonged to same module with other oxidative phosphorylation pathway module (**Fig. S6F**). While the deficiency of FAT1 is implicated in increased oxygen consumption and increased proliferations in smooth muscle cells^9^, the FAT1-mediated mitochondrial dys-regulation in epithelial cells is still unknown. In summary, the protein network models provide unique opportunities to systematically identify tumor interactomes beyond transcriptome-based models which cannot capture post-transcriptional effects^23^.

We also identified highly preserved protein modules across seven cancer types as pan-cancer protein modules (**Fig. 1F, G**). The network of cross-talks revealed several pathways commonly exploited in these cancers including mitochondrial oxidative phosphorylation (MOP), endoplasmic reticulum associated degradation (ERAD), and hemostasis. N-acetyltransferase activity and ribosome emerged as the bridging pathways between MOP and ERAD.

Within MOP, consistent down-regulation of the NADH dehydrogenase pathway (C3 and C9; **Fig. 1F**) across multiple cancers is in line with the observed reduction in Complex I of the respiratory chain in many cancer cells^24^. NADH dehydrogenase, also known as respiratory complex I, is an enzyme that converts nicotinamide adenine dinucleotide (NAD) from its reduced form (NADH) to its oxidized form (NAD+) and translocates protons across the inner mitochondrial membrane^25^. In cancer cells, the NAD+/NADH balance is often disrupted. In breast cancer, the reduction of NAD+ in tumor cells rendered more aggressive and increased metastasis, in contrast to the anti-invasive effect from the enhanced NAD+/NADH balance^25,26^.

ERAD ensures that only properly folded and assembled proteins are transported to their final destinations and is exploited in tumor cells, which are usually under tremendous stresses such as hypoxia and nutrient deprivation^27^. The very mechanism can also induce cell death via induction of pro-apoptotic transcription factors when cells are over-flooded with misfolded proteins (i.e., ER stress)^28^. There are several proteasome inhibitors such as bortezomib^28^ and Withaferin-A^29^ to induce such apoptotic ER stress in tumor cells. Further, Golgi homeostasis (from C16), an organelle that receives the processed proteins from ER, can be disrupted by the proteasome to induce apoptosis in cancer cells^31^.

C14 (N-acetyltransferase activity) contains several components of N-alpha-acetyltransferase complex (also known as NatA complex: *NAA10*, *NAA15* and *NAA50*) and their regulator *HYPK* (regulator of *NAA10-15*^30,31^) with *ANKRD17* (multi-functional regulator of cell cycle/DNA regulation^32^, innate immune defense against viruses^33^ and bateria^34^) and *G3BP2* (a scaffold protein that plays an essential role in cytoplasmic stress granule formation). *NAA10* has been extensively studied in cancer literature and has oncogenic or tumor-suppressive roles depending on cancer types^35^.

The NatA complex catalyzes the addition of acetyl groups at N-terminus of various proteins emerging from the ribosome, increases the diversity in the protein functions and has a broad substrate specificity to acetylate most peptides^36,37^. This explains the proximity of the ribosome complex in C20 representing the full extent of co-translational modification machinery. Loss of NatA enzymatic activity impairs mitochondrial degradation^38^, and N-terminal acetylation affects proteasome localization and cell fitness^39^, in alignment with closely interacting ERAD and MOP axes (**Fig. 1G**). Overall, its links to commonly over-exploited carcinogenic ERAD and MOP, and its capacity to modify a broad spectrum of proteins (80~90% of human proteins) affirm its central role in orchestrating oncogenic pathways in tumor cells.

Lastly, we systematically identified and validated several pan-cancer regulators across the seven cancer types. SMC2 (structural maintenance of chromosomes 2), a central component of the condensing complex, is a subunit of condensin I and II^40^, and SMC2-centered network showed strong interactions with SMC4, another SMC ATPase subunit that is shared in condensin I and II. The condensins are major regulators of chromosome condensation and segregation during cell cycles, and also play roles in cell cycle checkpoints, spindle assembly and kinetochore organization^40^.

Our loss-of-function experiments in lung, colon, kidney and breast cancer cells showed the strongest anti-tumor effects across all tested cancer cells. Indeed, SMC2 alterations have been implicated in pyothrax-associated lymphoma^41^, MYCN-amplified neuroblastoma^42^ and gastro-intestinal cancers^43^. As the major function of condensins are chromosome segregation, alterations in SMC2 induced genomic instability. Condensin-II depleted cells concurred defects in homologous recombinant (HR) DNA repair^44^. These were evident in SMC2-centered pan-cancer network model which captures several DNA repair regulators (e.g. - FANCI, UHRF1, LIG1, CHAF1B; **Fig. 2D**).

On the other hand, we also observed the DNMT1-UHRF1 axis in SMC2-centered network model, a key epigenetic mechanism for global DNA methylation maintenance and histone modifications^45^. While the pro-tumorigenic interplay between condensins and the DNMT1-UHRF1 axis is unknown, their interactions with a network neighbor, TOP2A (topoisomerase IIα, **Fig. 2D**), may serve as the common denominator to link the missing knowledge gap between the SMC2-mediated genomic stability and DNMT1-UHRF1 epigenetic axis^46,47^.

EZH2, catalytic core protein in the Polycomb Repressor Complex 2 (PRC2) that methylates H3K9me and H3K27me and acts as transcriptional repressor^48^, is an oncogene in a wide range of cancers and closely interacts with SMC2 in the protein network model (**Fig. 2D**). While their interactive mechanisms are unknown, condensins and EZH2 play crucial roles in forming DNA loops where condensins extrude the DNA^49^ and the PRC2 complex bends to form the DNA loop^50^. These suggest condensin-I/II-PRC2 axis constitutes a broader epigenetic mechanism to confer pro-tumorigenic regulations beyond cell cycle and genomic stability.

RSL1D1 (also known as cellular senescence-inhibited gene) is another top pan-cancer regulator and is up-regulated in multiple cancer types (**Fig. 2C**). Its over-expression promotes cell proliferation in HCC *in vitro* by activating c-MYC^51^, is associated with poor prognosis in prostate cancer^52^, and inhibits PTEN translation in replicative senescence in glioblastoma cells *in vitro*^53^. The protein co-expression network analysis indicates RSL1D1 is the hub and a key driver in M220 in CRC, which is enriched for hallmark MYC targets (cFET p=1.29E -6, 12.8 EFC), and suggesting it may modulate the oncogenic MYC activity in CRC.

DDX21 (DEAD/H-Box Helicase 12) acts as a sensor of the transcriptional status of both RNA polymerase (Pol) I and II, promoting ribosomal RNA (rRNA) processing and transcription from polymerase II (Pol II)^54,55^. While other DEAD box RNA helicases such as DDX1/3/6/5/17 are known to affect aberrant RNA export and translations in cancers in both of oncogenic and tumor-suppressive contexts^56^, the role of DDX21 remains mostly unknown. Therefore, DDX21 is an attractive novel pan-cancer target.

Interestingly, both RSL1D1- and DDX21-centered networks are enriched for rRNA biogenesis pathways in LUAD and STAD protein networks (**Fig. 2B, C**). PDCD11 (programmed cell death 11, essential for mature 18S rRNA generation) is one of the rRNA biogenesis protein, that is one of 147 targets emerged from CRISPR-Cas9 screens over 31 colorectal cancer cell lines^57^. PDCD11 is an NF-κβ binding protein that modulates NF-κβ pathway activity in CD8+ T-cells^58^. HEATR1 is another ribosome biogenesis factor that modulates cancer cell survival in non-small cell lung cancer^59^. Several *HEATR1* peptides can act as glioma-associated antigens that induce a cytotoxic T-cell response to target glioma stem-like cells^60^.

HEATR1 and PDCD11 interact closely with NAA25 (a non-catalytic subunit of the NatB complex which catalyzes acetylation of the N-terminal methionine residues) and a lung cancer driver mutation in *PIK3CA* (**Fig. S13A**). PIK3CA mutations are known to evade tumor immunity by promoting inhibitory myeloid environment^61^, and inhibition of the PI3K pathway is implicated in enhanced immune-therapeutic responses in many cancers^62,63^. However, the bridging mechanism to modulate anti-tumor immunity has not been understood yet. Our analyses suggest post-translational modification such as N-terminal acetylation by the NatB complex is a key modulator exploited in immune evasion in lung adenocarcinoma.

**MATERIALS AND METHODS**

*Data Collection*

Initially, we collected publicly available protein expression data sets. From *Clinical* Proteomic *Tumor Analysis Consortium* (CPTAC)^64^, we gathered protein expressions and respective clinical annotations for tumor and matched-normal samples for breast carcinoma (BRCA), clear renal cell carcinoma (CCRCC), colorectal carcinoma (CRC)^65^, hepatocellular carcinoma (CPTAC-HCC)^66^, lung adenocarcinoma (LUAD), ovarian carcinoma (OV) and uterine corpus endometrial carcinoma (UCEC). Additionally, we further obtained protein expressions and clinical data for HBV-infected HCC (HBV-HCC), and stomach cancer (STAD)^67^ from the literature.

Upon collection, we carried out a series of data quality control (QC) steps to filter out inadequate data for co-expression network analyses. Firstly, we performed data quality control (see *Data Quality Control for Network Analysis*) to adjust for confounding variables and batch effects. Then, we compared each cancer proteome with respective transcriptome to confirm if some of gene-gene interactions observed in the proteome are preserved. To do so, we constructed a set of preliminary protein co-expression networks and modules, and tested their preservations using Module Preservation analysis implemented in WGCNA R package^68^ with the suggested threshold of Bonferroni corrected p-value < 1E-10. Supposedly, we expected each cancer proteome bears substantial fraction of modules preserved in the transcriptome. To our surprise, none of CPTAC-HCC and MB exhibited preserved modules in the transcriptome and flagged out in the further analyses. This resulted in HBV-HCC to serve as the representative HCC proteome data set in the following analyses, thereon denoted as HCC.

Additionally, we required each cancer to provide at least one significant differentially expressed protein signature when contrasted to matched normal samples or across clinical cancer stages. This was necessary to address the dys-regulated proteomes in cancers. As a result, OV was further filtered out as it did not yield any differentially expressed protein signatures.

Overall, the data collection and QC yield seven cancers, BRCA, CCRCC, CRC, HCC, LUAD, STAD, and UCEC, for further analyses.

*Differentially Expressed Proteins (DEPs)*

We used MS-based proteomics data from CPTAC to identify differentially expressed proteins (DEPs) by leveraging available tumor and matched normal samples from the same patients. Overall, among the 7 CPTAC studies containing normal tissue samples (BRCA, CCRCC, CRC, HCC, LUAD, OV, UCEC), there were 415 tumor proteomes with an available matched-normal proteome. For each cancer cohort, we performed a tumor-vs-normal paired analysis to identify differentially expressed proteins (DEPs) by adjusting for the confounding variables and batch effects including age, gender, ethnicity, sequencing center, sequencing instrument, sequencing date and sequencing operator using limma^69^ implementation in R (v3.40.6).

*Gene Perturbation Signature Analysis*

In order to validate protein-protein (or mRNA-mRNA) interactions captured by protein co-expression networks (or gene co-expression networks), we tested if experimentally identified gene interactions were present in the networks. To do so, we utilized shRNA knock-down signatures in cancer cells, curated by the Library of Integrated Network-based Cellular Signatures (LINCS) database. The differential expressions in shRNA knock down or over-expressions of a target gene in transfected cancer cells capture interacting genes with the target perturbed genes, which were evaluated using *limma* R package^69^. These gene perturbation signatures were tested for enrichment in the network neighborhood of the target gene by Gene Set Enrichment Test (GSEA), implemented in the R package fgsea (v1.10.1). The network neighborhood captured the closely interacting genes and were systematically identified as the approachable genes by hopping *l* links from the target gene in a network, and we restricted *l* ≤ 2 and network neighborhood not to exceed 10% of the global network size. Overall, we were able to test gene perturbation signatures from cell lines matching for BRCA (MCF7: 3,726 genes), CCRCC (HA1E: 3,788 genes, HEK293T: 1,110 genes, HEKTE: 385 genes), CRC (HT29: 3,619 genes, SW480: 325 genes), HCC (HEPG2; 3,683 genes) and LUAD (A549: 3,735 genes, HCC515: 3,648 genes, SKL: 550 genes).

*Protein Expression Data Processing*

We used the MS-based proteomics data from CPTAC 2 and CPTAC 3 studies corresponding to breast cancer (BRCA), clear cell renal carcinoma (CCRCC), colorectal cancer (CRC), hepatocellular carcinoma (HCC), lung adenocarcinoma (LUAD), stomach adenocarcinoma (STAD), uterine corpus endometrial carcinoma (UCEC) cohorts^65,67,70-73^. Overall, there were 415 tumor proteomes with matched normal proteomes (see **Table S1** for details). For each cohort, we kept only one technical replicate with the most complete data and kept all biological replicates. In the HCC proteomes, we consolidated protein isoforms by using the R package **WGCNA** with ‘connectivity-based collapsing’ option^74^. We used the protein expressions, calculated as log(sample/reference) by multi-plexed iTRAQ or isobaric tandem mass tags (TMT) in the global MS experiments. By default, the expression ratios were further normalized so that all samples within a cohort had the Median Absolute Deviation (MAD) of 1.

*Differentially Expressed Proteins (DEPs) and Differentially Expressed Genes (DEGs)*

Differentially expressed proteins (DEPs) were identified by comparing matched tumor and normal samples in the paired test. Among the 6 CPTAC studies containing normal tissue samples (BRCA, CCRCC, CRC, HCC, LUAD, UCEC), 415 tumor proteomes had matched normal proteomes. For STAD, the samples lacked matched normal samples, and we resorted to DEPs between Epstein Barr virus (EBV) + and EBV- samples as a proxy for distinctive tumor signatures^15^. For each cancer type, we compared the tumors and the normal samples to identify DEPs (tumor-DEPs) while adjusting for the confounding variables and batch effects including age, gender, ethnicity, sequencing center, sequencing instrument, sequencing date and sequencing operator using the R package **limma^69^** (v3.40.6). We also identified proteins specifically differentially-expressed for each cancer type as estimated by the difference in the protein abundance level, $Specificity= FC_{cancer}- mean\left( FC_{other cancers} \right)$.

The normalized, batch and platform corrected RNA-seq data were obtained from the PanCancer Atlas consortium (https://gdc.cancer.gov/about-data/publications/pancanatlas). For each cancer type, lowly expressed genes were removed if their expressions are absent from more than 75% of samples. To identify DEGs, primary tumor samples (type code ”01A”) were compared to the normal tissues (type code “11A”) in each cancer type. Then the gene expression data were log2 transformed and fitted to a linear model, to adjust covariates including age, gender, race, TSS, portion, plate, and center. Moderated t-statistics, moderated F-statistic, and log-odds of differential expression were computed by **limma** (v3.4) using empirical Bayes statistics**^69^**. Significant DEGs between tumor and normal tissues were defined as fold change higher than 2 and adjusted *p* value less than 0.05.

*Data Quality Control for Network analysis*

Before the protein co-expression network analysis, we further performed comprehensive quality control on the normalized and filtered protein expression data. Firstly, we removed outlier samples by the principal component analysis (we considered the 1^st^ and 2^nd^ principal components, samples ≥ 4 standard deviations). Then, proteins expressed in less than 20 samples were removed, followed by imputation of the missing protein expressions by the k-nearest neighbor algorithm by *impute.knn()* in the R package **impute** (v1.58.0). Further, the data were adjusted for batch variables (data generating center, date, and machine) as well as biological confounders such as age, ethnicity, and gender by modeling via generalized linear model (*glm()* in **stats** R package, v4_3.6.1).

*Protein/Gene Co-expression Network Analysis*

The protein co-expression networks were identified by using Multi-scale Embedded Gene Co-expression Network Analysis (MEGENA)^75-77^. Briefly, MEGENA first selects protein pairs with significant correlations (FDR < 0.05) by sample permutation. Although filtered by statistical significance, the false positive pairs still dominate the co-expression network^77,78^. To handle this, MEGENA imposes a geometrical constraint by embedding onto a 3-dimensional topological sphere.^79^ The resulting co-expression network belongs to a class of sparse geometrical networks called “planar filtered networks (PFNs)”. PFNs can be drawn on the surface of the sphere without any link intersections.^80^

PFNs bear a broad spectrum of topological features of real-world complex networks such as scale-free degree distributions and small-world^81^. PFNs then go through unsupervised clustering for identification of network clusters (*i.e.,* gene modules) at various compactness scales.^75-77^ The resulting gene modules have hierarchical relationship. The hierarchy represents the multi-scale organization of gene modules with different degrees of compactness. It captures a series of relationships of higher-order (i.e., parent) modules possessing children modules residing within these parent modules. Children modules are more compact than their parent modules.^77^ Furthermore, candidate key drivers of gene modules are further identified by means of statistically significant hubs with hub p-value < 0.05.^77^ With module size threshold 50 ≤ |m_i_| ≤ 3000, MEGENA yielded the following number of modules including parent and child modules in **Table S4**.

Similarly, we performed MEGENA to construct a mRNA co-expression network for each cancer type using the respective pan-cancer transcriptome data from The Cancer Genome Atlast (TCGA). We first downloaded batch corrected gene expression data from TCGA Pan-Cancer Atlas initiatives (https://gdc.cancer.gov/about-data/publications/pancanatlas), and further performed quality control by excluding outliers (samples with four standard deviations away in the 1^st^ and 2^nd^ principal component space), and correcting for biological confounders such as age and gender. The corrected data were then provided as the inputs for MEGENA, and the same module size thresholds were applied as the protein networks, yielding the numbers of modules as in **Table S4**.

*Module Preservation Analysis between Each Cancer’s Proteome and Transcriptome*

For each cancer type, individual co-expressed protein modules were tested for preservation of its network connectivity in the transcriptome of the same cancer type, or in the proteome from a different cancer type by the module preservation analysis^68^. For all module preservation analyses, we utilized the implementation in **WGCNA** R package via *modulePreservation()* function (v1.68). The protein modules were deemed significantly preserved if Bonferroni corrected p-value < 1E-10 as recommended by the authors^68^. On the other hand, the protein module was deemed absent in the comparison if the summary z-score < 2^68^. Based on these thresholds, per a cancer type, a protein module was categorized into ‘proteome-specific’ if the summary Z-score < 2, or ‘transcriptome-preserved’ if the Bonferroni corrected p-value < 1E-10 from the module preservation analysis with the same cancer transcriptome. Similarly, a protein module was deemed “cross-cancer preserved” in a different cancer’s proteome if the Bonferroni corrected p-value < 1E-10, or deemed “not cross-cancer preserved” if summary Z-score < 2 when compared with another cancer’s proteome.

We collected the transcriptome cohorts for the seven cancer cohorts from CPTAC and TCGA Pan-cancer atlas cohorts. While the CPTAC consortium provides the patient matched transcriptome for five cancer types (BRCA, CCRCC, CRC, LUAD and UCEC), HCC and STAD were missing the matched transcriptome data, hence the direct comparison of proteome and transcriptome for all cancer types were not possible. On the other hand, TCGA Pan-cancer atlas transcriptome cohorts provided greater number of samples for all cancer types. To this end, we evaluated the concordance of the module preservation results from the TCGA Pan-cancer atlas and CPTAC cohorts. Specifically, we examined how the protein modules were preserved in the TCGA and CPTAC transcriptomic data (**Fig. S15**). We observed that the preservation z-scores, the key statistics for accessing module preservation, were highly concordant between TCGA pan-cancer atlas and CPTAC (**Fig. S15**), where a large number of transcriptomically preserved, or proteome-specific modules were detected from using TCGA pan-cancer atlas transcriptome data (**Fig. S15**). Overall, these results indicate the greater numbers of samples from TCGA pan-cancer atlas transcriptome cohort show greater benefits, hence they were used for the module preservation analysis.

*Pan-cancer Protein Interaction Communities (PCPIC) Analysis: Conserved protein modules across multiple cancer types*

We evaluated robustly co-expressed protein modules across different cancer types (see the workflow in **Fig. S7**). For each cancer type, we examined if the co-expressed protein modules are conserved in different cancer types. Given two co-expressed protein modules, M_i_^(n)^ from the n^th^ cancer type and M_j_^(m)^ from the m^th^ cancer type, we evaluate their conservation based on two criteria: i) *Correlation preservation* by evaluating if the protein expression profiles of the proteins in M_i_^(n)^ in the m^th^ cancer type are significantly correlated and those of M_j_^(m)^ in the n^th^ cancer type (i.e. *n ≠ m*) are correlated. ii) *Module overlap* by evaluating if there is a significant overlap between M_i_^(n)^ and M_j_^(m)^ through Fisher’s Exact Test (FET) with a cutoff of 0.05 for FET p-value (**Fig. S7A**). M_i_^(n)^ and M_j_^(m)^, are claimed to share a conserved proteome if they satisfy the criteria i) and ii).

We illustrate the conservation analysis workflow with an example which compares one module from breast cancer proteome, M_1_^(BRCA)^, to another from colorectal proteome, M_2_^(CRC)^ (**Fig. S7B**).

Criterion 1: We examine if the proteins in M_1_^(BRCA)^ or M_2_^(CRC)^ are also correlated in colorectal or breast cancer proteome, respectively. By utilizing module preservation analysis implemented in **WGCNA** R package via *modulePreservation()* function (v1.68), the proteins in the modules are deemed significantly correlated in the respective cancer type if Bonferroni corrected p-value < 1E-10 (a recommended p-value threshold by Langfelder *et al.* 2011^68^).

Criterion 2: We evaluate if M_1_^(BRCA)^ and M_2_^(CRC)^ show significant overlap by Fisher’s Exact Test (FET) with odds ratio (OR) > 1. If M_1_^(BRCA)^ and M_2_^(CRC)^ (or M2 is enriched in M1) with FDR adjusted FET p-value < 0.05, and vice versa, then the overlap is deemed significant, thus satisfying ii).

M_1_^(BRCA)^ and M_2_^(CRC)^ are conserved in breast and colorectal cancers if the criteria 1 and 2 are satisfied.

While the aforementioned workflow identifies pairs of modules, each pair represents shared protein interactions in two cancer types only. On the other hand, these pairs can collectively form a network of conserved modules which represents a set of conserved protein interactions across multiple cancer types (**Fig. S7B**). To this end, we systematically identified these tightly connected groups of modules by applying a graph-theoretic community detection algorithm, walktrap. We utilized its implementation in *cluster_walktrap()* function from igraph R package (v1.2.4.1). As results, we identified 20 highly conserved, pan-cancer protein interaction communities (PCPICs).

Within each PCPIC, proteins involved in key roles are often associated with higher connectivity and may reflect the crucial biological functions and pathways in cancer^77,82,83^. Thus, we identified a highly connected subnetwork of proteins within each PCPIC. Specifically, we constructed the union co-expression network of proteins within each PIC across the seven cancer types, then calculated the *coreness* of the proteins (the top plot in **Fig. S7C**). Coreness is defined through k-core, a subnetwork in which each node is connected with at least k other vertices (the bottom plot in **Fig. S7C**). Then, the coreness of a node is k, if the node belongs to k-core, but not to (k+1)-core^84^. In other words, a group of nodes (i.e., proteins) in the union network with maximal k-core represents the most connected subnetwork of the PCPIC. These PCPIC cores were utilized to understand the key biological functions as summarized in **Fig. S8** as enriched Gene Ontology (GO) terms and curated pathways.

*Pan-cancer Interactions Among Conserved Pathways*: While the PCPIC cores represent distinct protein interactions conserved across the seven cancers, we posit that there are higher-level interactions among them, i.e. cross-talks among the pan-cancer conserved pathways. We developed a framework to analyze these cross-talks among the PCPIC cores in the cancer proteome.

To evaluate such cross-talks, we first calculated the overall activity score of each PCPIC core by Gene Set Variation Analysis (GSVA; **Fig. S7D**-**(i)**)^14^. GSVA calculates the z-score for each sample for a given input signature, leading to an overall activation (z-score > 0) or a suppression (z-score < 0) within each sample’s proteome. Putting the z-scores together across all PCPICs and sample, it yields a data matrix where each feature is a summarized GSVA z-score for each PCPIC across all samples (**Fig. S7**-**(ii)**). Then, we evaluated interactions among the PCPICs by the pairwise Spearman’s correlations on the GSVA z-score matrix (workflow in **Fig. S7**, (ii); cancer type-wise results in **Fig. S7B**). The significant interactions were identified by FDR < 0.05.

Then, we summarized which protein interactions were robustly observed across different cancer types. Specifically, for each pair of PCPICs, we counted the number of cancer types, in which several pairs of PCPICs showed positive or negative correlations consistently across at least four cancers, forming a robust network (**Fig. S7C**).

*Module Prioritization*

For each cancer, the co-expressed protein modules were evaluated for enrichment of differentially expressed proteins (DEPs) in tumor versus adjacent normal, cancer driver mutations and pan-cancer driver mutations by Fisher’s Exact Test (FET). These FET p-values for each module were then summarized into a module significance score, *MS*, defined as:

$$MS=\sum_{i=1}^{N} \frac{{-log}_{10} ({FET P(m, PS}_{i})}{N}$$

where *m* refers to a module, *PS* represents a set of protein signatures, N is the number of tests, *P(m, PS_i_)* is Fisher’s Exact Test p-value for enrichment of the *i*^th^ protein signature in the module m.

*Key Driver Analysis (KDA) and Top Regulator Nominations*

We hypothesized the key regulators of pro-tumorigenic pathways closely interact with dys-regulated pathways in cancer proteomes. To this end, we treated the differentially expressed proteins (DEPs) in tumor samples as the protein signatures of such dys-regulated pathways and searched for protein nodes interacting with DEPs in the co-expression networks as the potential pro-tumorigenic regulators. Such potential protein drivers were identified by Key Driver Analysis (KDA)^85^. Firstly, for a given protein we identified its neighbors in the network which are within 3-layer layers away from the protein. Then, we evaluated the enrichment of the DEPs in the neighborhood by performing Fisher’s Exact Test (FET) and determined a protein as the potential pro-tumorigenic regulator if the FDR adjusted FET p-value is smaller than 0.05^86^.

We used more DEPs based on the more relaxed thresholds (i.e., limma FDR < 0.15, |FC| > 2) for the KDA so as to capture the weaker signals that may have been overlooked by the stringent FDR < 0.05 threshold. As there was no respective normal proteome data for STAD, we used DEPs in Epstein-Barr virus (EBV) infected samples, which are known to drive tumorigenesis in stomach cancer^87^.

Then, we calculated the Pan-cancer regulator score to prioritize the predicted key regulators. Among the seven cancer types, we counted: i) the number of cancer types in which each gene is a hub with hub p-value < 0.05, *N_hub_*, ii) the number of cancer types in which each gene’s 3-layer neighborhood significantly enriched for DEP signatures, *N_enrich_*, and iii) the number of cancer types in which each gene is a DEP signature, *N_DEP_*. Then, the sum of these three scores, S_pan-cancer_= *N_hub_+ N_enrich_ + N_DEP_*, was utilized the pan-cancer regulator score.

The prioritized list was further filtered to nominate novel pan-cancer regulators. To this end, we sought to identify pan-cancer regulators of up-regulated pathways in tumors, and are relatively under-studied in the literature. The up-regulated regulators were identified with number cancer types in which each gene is an up-regulated DEP, *N_DEP-UP_* > 4, with no hits from down-regulated DEPs, *N_DEP-DN_* = 0. Using easyPubmed (v2.13) R package, we also queried the Pubmed with “cancer” and gene symbol name for each protein, and restricted those with less than 50 known studies. The filtered list highlights top three proteins that proceeded for experimental validations**.**

***Experimental procedure and method***

*Lentivector Production*

HEK293T cells were cultured in a T150 flask in 20mL complete medium [DMEM (Corning #10-013-CV), 10% FBS (Gibco #16000-044), 100u/ML Pen-Strep (Gibco #15140-122)] to no greater than 90% confluence, plated in 100mm Petri dishes at a density of 7x10^6^ cells/dish, and allowed to grow overnight at 37°C, 5% CO_2_, 95% RH. Mission shRNA 3^rd^ generation lentivector bacterial stocks (Sigma #05282119MN: shDDX21 clone TRCN0000051200, shRSL1D1 clone TRCN0000159162, shSMC2 clone TRCN0000062538) and Mission pLKO.1-Puro Non-Mammalian shRNA Control (Sigma #SHC002) were cultured overnight in Miller LB-100μg/mL carbenicillin (Fisher #BP1426-2 and #BP2648-5) medium, and plasmids were isolated using the QIAprep Spin Miniprep Kit (Qiagen #27106) as described in the protocol. Two hours prior to transfection, the medium on the production plates was changed to 10mL serum- and antibiotic-free OptiMEM (Gibco #31985-070). Transfection was performed using Lipofectamine 3000 transfection reagent (Invitrogen #L3000-015), 7.5μg of vector plasmid, 1.5μg pMD2.G (Addgene #12259), 2.5μg pMDLg/pRRE (Addgene #12251), and 1.25μg pRSV-Rev (Addgene #12253). Reagent and plasmid dilutions were each performed in 500μL OptiMEM, combined per the protocol instructions, and the completed mixtures were added dropwise to the production plates. Transfected cells were incubated for four hours, followed by a medium change to 12mL transduction medium [OptiMEM, 5% FBS, 1mM sodium pyruvate (Gibco #11360-070)]. Transfected cells were cultured for 48 hours, after which the medium was drawn off the production plates and centrifuged for 10 minutes at 800rcf and 4°C. Supernatant was drawn off and transferred to fresh 15mL conical tubes containing one part 4x lentivirus concentration buffer to 3 parts viral supernatant (~3-4mL), mixed thoroughly, and incubated overnight on nutating mixer at 4°C. Viral supernatant and concentrator mixtures were centrifuged for one hour at 1600rcf and 4°C, after which the supernatants were aspirated. Pellets were resuspended in 400μL PBS (Corning #21-040-CV), divided into 50μL aliquots, and stored at -80°C until needed.

*shRNA Knockdown and Growth Assay*

Gene knockdown was attained by seeding 5x10^5^ cells/well each of HEK293T, A498, H847, HCT116, and MDA-MB-231 into 6-well plates with 1:200 dilutions of Mission shRNA lentivectors in 2mL/well transduction medium-10μM Polybrene (AmericanBio #AB01643) with overnight incubation, followed by 48 hours’ culture in puromycin selective medium [DMEM, 10% FBS, 100 U/mL pen-strep, 10μg/mL puromycin (Sigma #P8833)]. After 48 hours, transduced cells were passaged and plated into the central 60 wells of 96-well plates in 200μL complete medium at a seeding density of 4000 cells/well, six wells per treatment condition (untransduced control, non-targeting shRNA control, shDDX21, shRSL1D1, and shSMC2), two parental lines per plate. Surplus cells were cultured in 10mL puromycin selective medium in T75 flasks and allowed to expand for additional assays. Plates were imaged six hours after plating (T = 0) and then daily for four days (days 1-4) to assess confluency using the Perkin Elmer Operetta CLS imaging system. Percent confluency per well was averaged for each treatment condition and timepoint, standard deviations were calculated, and averages for each parental cell line were normalized to the appropriate non-targeting shRNA control. After four days, total cell viability was assessed using the CellTiter-Glo Luminescent Cell Viability Assay (Promega #G7570) as described in the protocol,^^[[1]](#endnote-1)^^ and luminescence signal was measured using the Tecan Infinite M200 plate reader with 1 second integration time. Average background luminescence was subtracted from the luminescence value for each well, luminescence signals were averaged for each treatment condition, standard deviations were calculated, and averages for each parental cell line were normalized to the appropriate non-targeting control.

*Confirmatory qPCR*

Total RNA was isolated using the RNeasy Mini Kit (Qiagen #74106) as described in the protocol. Approximately 1x10^5^ cells of each parental line and treatment condition were reserved and lysed in Qiagen RLT buffer with 1% β-mercaptoethanol (Fisher #O3446I). Single-strand cDNA was generated from total RNA isolates with the SuperScript First-Strand Synthesis System for RT-PCR (Invitrogen #11904018), as described in the protocol using the included oligo(dT)_12-18_ primers. Quantitative PCR was performed for all conditions, in triplicate 10μL/well reactions in a 384-well PCR plate, using PowerUp SYBR Green Master Mix (Applied Biosystems #A25742) and KiCqStart SYBR Green Primer sets (Sigma #KSPQ12012G) against GAPDH (H_GAPDH_1), DDX21 (H_DDX21_1), RSL1D1 (H_RSL1D1_1), and SMC2 (H_SMC2_2) on the ViiA 7 Real-Time PCR System (Applied Biosystems), as described in the master mix protocol. Relative gene expression was quantified and graphed via the native ΔΔ_CT_ analysis method in the ABI QuantStudio Real-Time PCR Software suite (Applied Biosystems, version 1.3), with each cell type normalized to its untransduced parental control using GAPDH as reference gene.

**SUPPLEMENTAL TABLES**

Table S1. Cohort descriptions of cancer proteome data sets.

| **Cancer Type** | **Breast Cancer** | **Clear Cell Renal Cell Carcinoma** | **Colorectal Cancer** | **Lung Adenocarcinoma** | **Early Onset Gastric Cancer** | **Uterine Corpus Endometrial Carcinoma** | **HBV-related Hepatocellular Carcinoma** |
| --- | --- | --- | --- | --- | --- | --- | --- |
| **Abbreviation** | **BRCA** | **CCRCC** | **CRC** | **LUAD** | **STAD** | **UCEC** | **HCC** |
| **Data Source** | CPTAC BRCA Confirmatory Study | CPTAC CCRCC Discovery Study | CPTAC Colon Cancer Confirmatory Study | CPTAC LUAD Discovery Study | CPTAC Proteogenomics of Gastric Cancer | CPTAC UCEC Discovery Study | CPTAC HBV-Related HCC |
| **Sample Size (Tumors/Normals)** | T: 115  N: 18 | T: 110  N: 84 | T: 95  N: 100 | T: 109  N: 102 | T: 80  N: 80 | T: 97  N: 20 | T: 159  N: 159 |
| **Female Percentage** | 100% | 25.2% | 57.4% | 34.6% | 55% | 100% | 19.5% |
| **Average Age at Onset** | 60.4 | 60.6 | 65.2 | 62.7 | 37.0 | 63.7 | 53.7 |
| **Tumor Stage Distribution (TNM)** | 1: 3.0% 2: 60.4% 3: 26.9%  NA: 9.7% | 1: 41.8% 2: 15.5% 3: 34.5%  4: 8.2% | 1: 10.2% 2: 40.6% 3: 41.1%  4: 8.1% | 1: 53.5% 2: 27.5% 3: 18.5%  4: 0.5% | NA: 100% | 1: 76.1% 2: 6.8% 3: 14.5%  4: 2.6% | 1: 57.2 2: 8.8 3: 32.7 4: 1.3 |

Table S2. Summary of differentially expressed protein (DEP) signatures (FDR < 0.05, fold change > 2 or < ½).

| **Signature Type** | **Direction** | **BRCA** | **CCRCC** | **CRC** | **HCC** | **LUAD** | **STAD** | **UCEC** |
| --- | --- | --- | --- | --- | --- | --- | --- | --- |
| **Tumor vs normal DEP** | up-regulated | 45 | 2,871 | 477 | 1,728 | 2,852 |  | 2,658 |
|  | down-regulated | 138 | 2,706 | 673 | 1,700 | 2,885 |  | 2,394 |
| EBV infected vs **rest** | up-regulated |  |  |  |  |  | 16 |  |

Table S3. Number of differentially expressed proteins (DEPs) in the tumor proteome in each cancer type and the number of DEPs without differential expression at the mRNA level in the respective tumor transcriptome. DEPs were determined by FDR < 0.05 while not differentially expressed genes (notDEGs) were identified by FDR > 0.2 (see Materials and Methods for details).

| **Cancer Type** | **Total #. Proteins** | **#. DEPs** | **#. DEPs but not DEGs** |
| --- | --- | --- | --- |
| **BRCA** | 10,468 | 45 | 8 |
| **CCRCC** | 9,964 | 2,871 | 830 |
| **CRC** | 7,418 | 477 | 59 |
| **HCC** | 6,478 | 1,728 | 747 |
| **LUAD** | 11,029 | 2,852 | 1,301 |
| **UCEC** | 10,768 | 2,658 | 786 |
| **STAD** | Matched normal proteome data is missing for DEP analysis | | |

Table S4. Numbers of protein and mRNA coexpression modules in seven cancer types.

| **Cancer** | **#. Protein Modules** | **#. mRNA Modules**  **(PanCanAtlas)** | **#. mRNA Modules**  **(CPTAC)** |
| --- | --- | --- | --- |
| BRCA | 134 | 242 | 159 |
| CCRCC | 138 | 248 | 158 |
| CRC | 99 | 233 | 172 |
| HCC | 83 | 237 | N/A |
| LUAD | 155 | 244 | 171 |
| STAD | 122 | 272 | N/A |
| UCEC | 15 | 212 | 143 |

Table S5. List of core proteins in the pan-cancer protein interaction communities (PCPICs) .

| **PCPIC** | **Core Proteins** |
| --- | --- |
| ***C1*** | SPTA1, ANK1, EPB42, HBA1, HBB, CA1, SLC4A1, SPTB |
| ***C2*** | PSMD1, PSMC2, PSMD2, PSMC4, PSMC5, PSMD6, PSMD12, PSMD3, PSMD11, PSMD13, PSMC1, PSMD8 |
| ***C3*** | NDUFS1, NDUFB11, NDUFV1, NDUFA12, NDUFA10, NDUFB5, NDUFS5, NDUFS4, NDUFA9, NDUFS8, NDUFS6, NDUFB3, NDUFS7, NDUFS3, NDUFA13, NDUFB1, NDUFA8, NDUFA5, NDUFB10, NDUFB7, NDUFB6, NDUFB4, NDUFA7, NDUFA11, NDUFS2, NDUFC2, NDUFB9, NDUFB8, NDUFV2 |
| ***C4*** | MRPL17, MRPL39, MRPL15, MRPL19, MRPL28, MRPL37, MRPL46, MRPL11, MRPL3, MRPL44, MRPL16, MRPL20, MRPL10, MRPL47, MRPL13, MRPL4, MRPL21, MRPL24, MRPL18, MRPL1, MRPL45, MRPL12, MRPL2, MRPL40, MRPL23, MRPL30, MRPL38, MRPL55, GADD45GIP1, MRPL54, MRPL41, MRPL50, MRPL58, MRPL48, MRPL49, MRPL53, MRPL9 |
| ***C5*** | IFIT1, OAS2, ISG15, MX1, CMPK2, IFIT3 |
| ***C6*** | MRPS31, MRPS22, MRPS23, MRPS2, MRPS21, MRPS28, MRPS27, MRPS17, MRPS15, MRPS33, MRPS11, MRPS26, MRPS9, MRPS25, MRPS12, MRPS5, EARS2, MRPS34, PTCD3, MRPS7, MRPS16, MRPS6 |
| ***C7*** | INTS1, INTS3, INTS2, INTS4, INTS7, INTS10, INTS8 |
| ***C8*** | PSMA4, PSMA2, PSMA5, PSMA6, PSMA3, PSMB1, PSMB3, PSMA1, PSMA7, PSMB4, PSMB8 |
| ***C9*** | NDUFB2, NDUFB4, NDUFB3, NDUFA13, NDUFB8, NDUFB9, NDUFC2 |
| ***C10*** | COX4I1, COX2, COX5A, COX5B, COX7A2L, COX7A2, COX7B, COX6C, COX7C, NDUFA4, COX6B1 |
| ***C11*** | THOC1, THOC2, THOC5, THOC3, THOC7, THOC6 |
| ***C12*** | CCT2, CCT5, CCT4, CCT6A, CCT3, CCT8, CCT7, TCP1 |
| ***C13*** | CALR, PDIA4, DNAJB11, HSP90B1, MANF, PDIA6, HYOU1, ERP44, GANAB, HSPA5, PDIA3, DNAJC3, MESD, MYDGF, P4HB, CNPY2, PRKCSH, PRDX4 |
| ***C14*** | NAA10, NAA15, ANKRD17, HYPK, NAA50, G3BP2 |
| ***C15*** | MRPS15, MRPS22, DAP3, MRPS5, MRPS31, MRPS26, MRPS35, LRPPRC, MRPS17, MRPS2, MRPS27, MRPS33, MRPS25, MTIF2, MRPS9, MRPS6, MRPS7, PTCD3 |
| ***C16*** | COG1, COG2, COG5, COG3, COG4, COG7, COG8, COG6 |
| ***C17*** | COMMD7, CCDC22, CCDC93, COMMD10, COMMD5, FAM45A |
| ***C18*** | IGHG1, IGLL5, IGLL1, IGKV1-12, TGIF1, IGLV1-40, JCHAIN |
| ***C19*** | FGB, FGA, C8A, C8B, F2, C6, ITIH1, C5, A1BG, KLKB1, CFH, CPN1, C9, AMBP, GC, ITIH2, PLG, ITIH4, ALB, APOA1, APOH, KNG1, CPN2, C8G, HPX, AHSG, ITIH3, PROS1, APOC1, APOM, FGG, APOA2, SERPING1, TF |
| ***C20*** | RPL4, RPL3, RPL6, RPL7A, RPL27, RPL7, RPL13, RPL28, RPL8, RPL24, RPL13A, RPL21, RPL23A, RPL19, RPL17, RPL18A, RPL14, RPL15, RPL18, RPS6, RPS8, RPL36AL, RPL34 |

SUPPLEMENTAL FIGURES


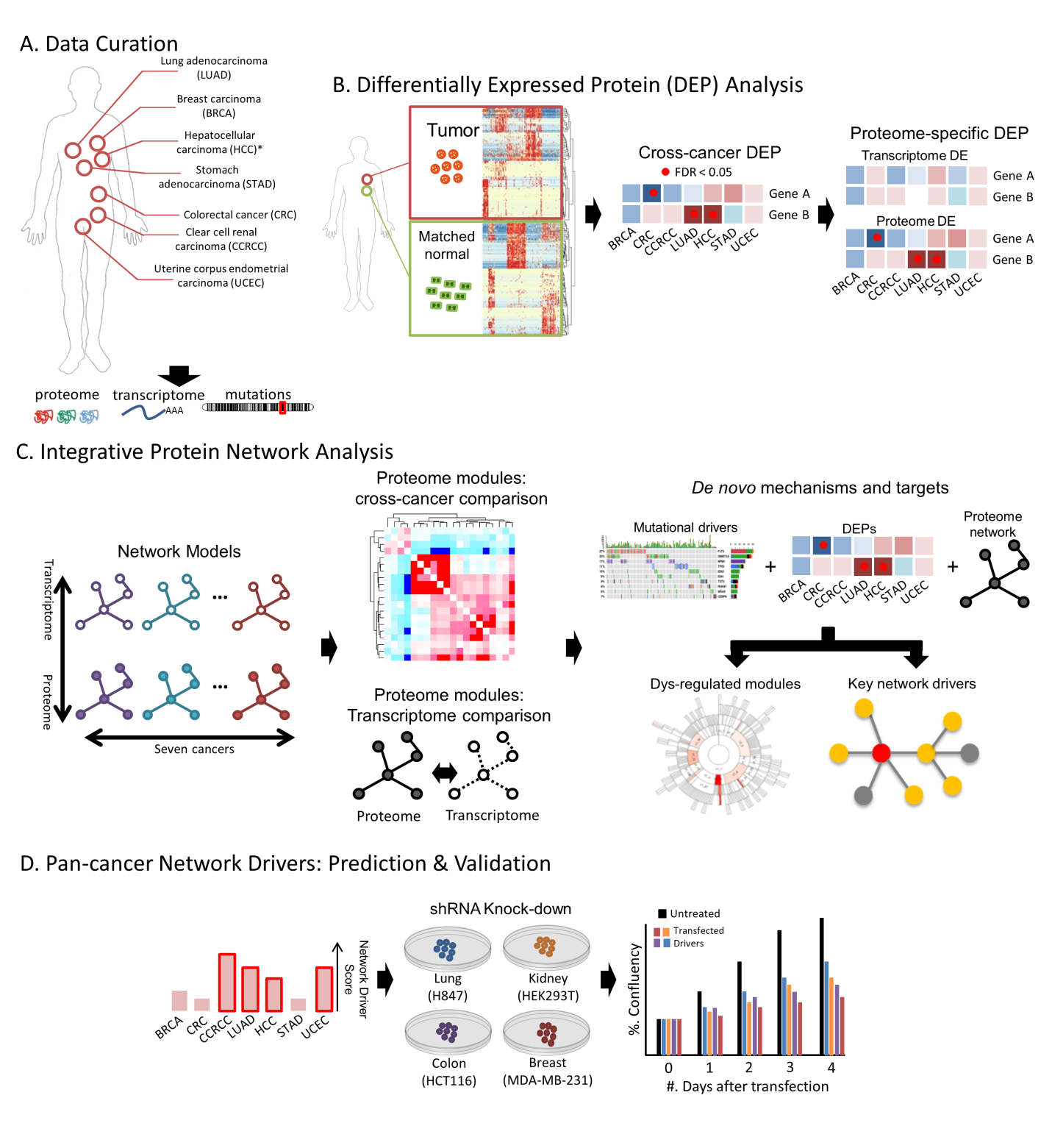


Fig. S1. The overall workflow of seven cancer proteome network analysis. A. Data curation: The figure illustrates the seven cancer types studied in this study, and data types utilized (proteome, transcriptome and mutation). B. Differentially Expressed Protein (DEP) analysis: Differentially expressed proteins (DEPs) and transcripts are identified by comparing tumor and matched normal samples per cancer type (in i). The DEPs from each cancer type are examined for differential expressions in other cancer types (in ii), or in respective transcriptome for the same cancer type (in iii). C. Integrative Protein Network Analysis: Within each cancer type, co-expression networks are constructed for respective proteome and transcriptome (in i). The co-expressed proteome modules are compared across the seven cancers (in ii) to characterize cross-cancer preserved proteome modules, or against the respective transcriptome (in iii) to identify proteome-specific modules. Then, mutational drivers and DEPs are projected to proteome network to identify dys-regulated modules, and key network drivers of these molecular signatures (in iii). D. Pan-cancer network driver prediction and validation: From the key network driver analysis in C-iii, the network driver scores are collected across the cancer types to predict the pan-cancer network drivers (in i). shRNA knock-down is performed on the predicted drivers across different cells (in ii), namely, lung cancer (H847), fetal kidney (HEK293T), colon cancer (HCT116) and breast cancer (MDA-MB-231). Per cancer type, the confluency of shRNA-transfected cells are compared against untreated controls to examine the anti-tumor effects (in iii).


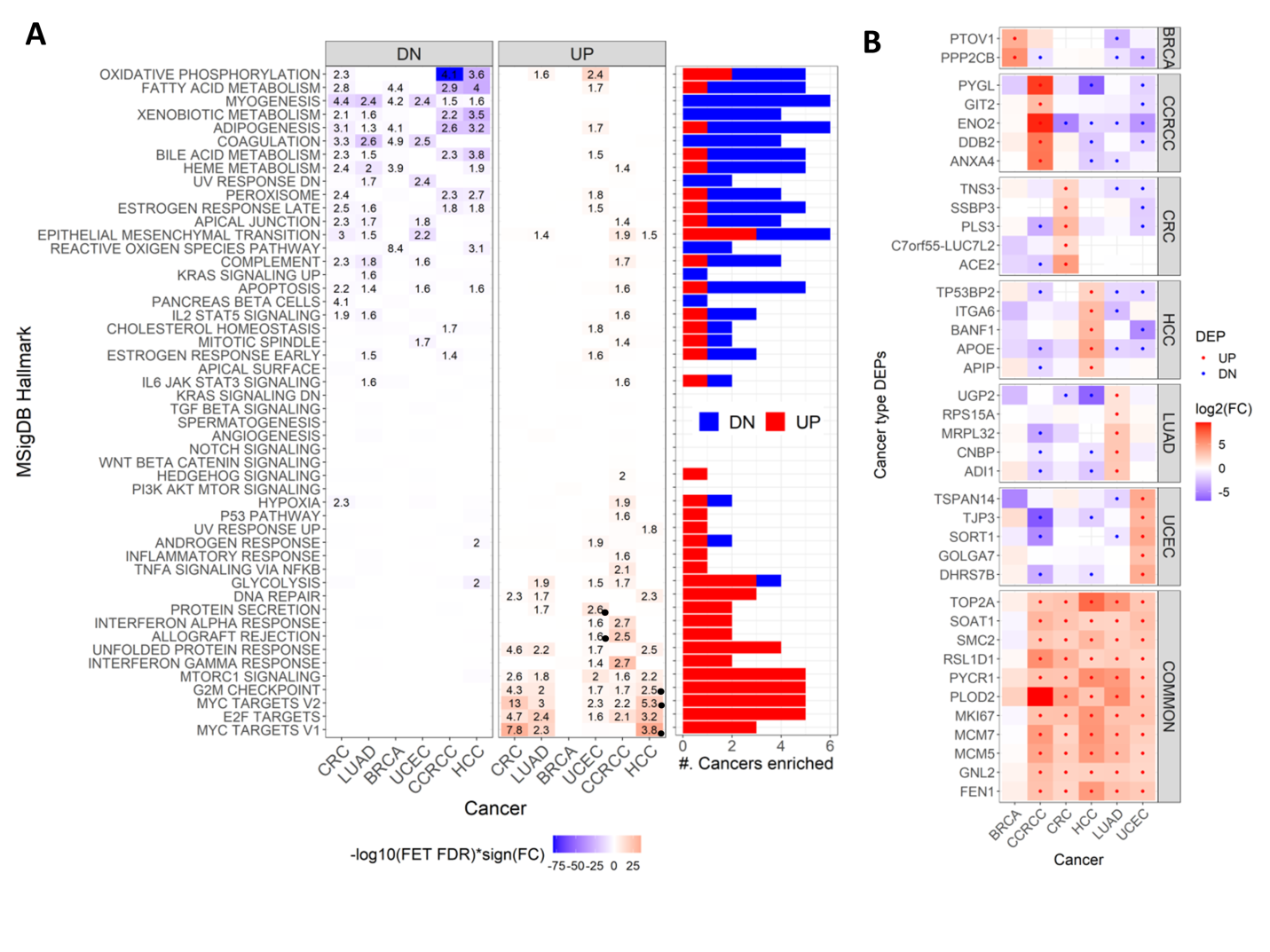


Fig. S2. A. Hallmark pathways enriched in the DEP signatures. The heatmap shows –log10(FDR corrected FET p-value) in blue color scale for down-regulated DEPs (left), and red color scales for up-regulated DEPs (center). Black dots in the heatmap marks pathways enriched by proteome-specific DEPs. The number of cancer types whose DEPs are enriched for the respective hallmark signatures is summarized in the barplot (right). B. The Cancer type-specific DEPs (marked as BRCA, CCRCC, CRC, HCC, LUAD and UCEC) and shared DEPs across multiple cancer types (marked COMMON). The cancer type combinations are marked at the bottom. The top 11 proteins commonly up-regulated across different cancer types are highlighted in the heatmap.

**
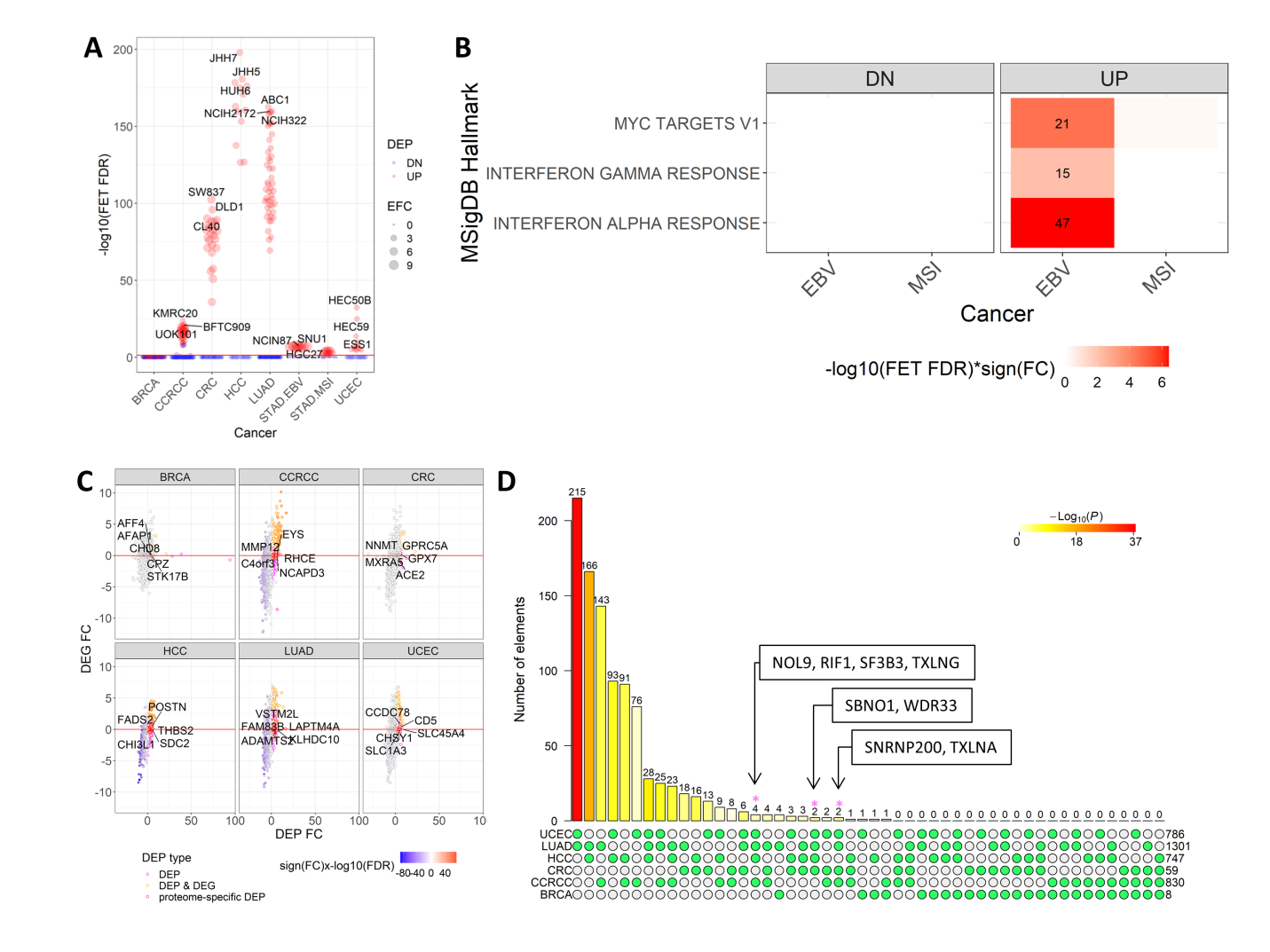
**

Fig. S3. Overview of differentially expressed proteins. A. Enrichment of cancer essential genes in differentially expressed protein signatures. Each dot represents enrichments of essential genes identified from each cancer cell line by CRISPRi screening in Archilles database (FDR < 0.05), with differentially expressed proteins (DEPs) in tumors compared to matched normal. For STAD lacking the matched normal samples, DEPs were obtained by comparing EBV+ samples to EBV- samples. B. Enriched hallmark pathways in differentially expressed proteins in EBV+ samples from STAD cohort. The heatmap shows –log10(FDR corrected FET p-value), representing enriched cancer hallmark signatures curated by MSigDB^1^. The numbers in the heatmap show enrichment fold-changes. C. Volcano plot of log2(fold change) between tumor and matched normal in proteome (x-axis), and transcriptome (y-axis). Differentially expressed DEPs in both proteome and transcriptome are colored yellow, and proteome-specific DEPs are colored in red. Top 5 proteome-specific DEPs with highest fold changes are highlighted. D. Pan-cancer proteome-specific DEPs: The barplot shows proteome-specific DEPs overlapping across different combinations of cancer types, with the heatmap color depicting the statistical significance by Super Exact Test (SET). The most recurrent proteome-specific DEPs (appearing across 4 cancer types) are highlighted.


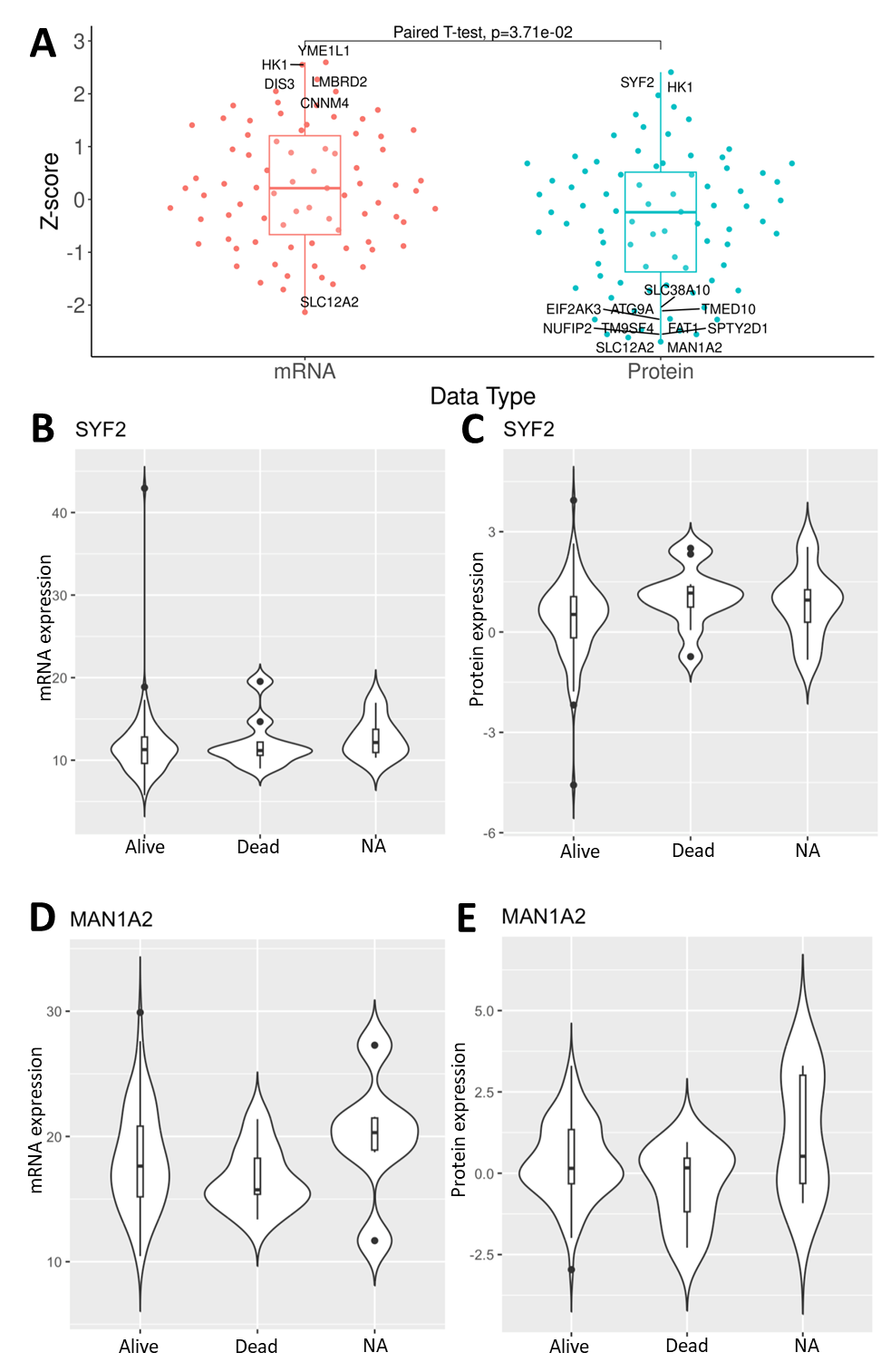


**Fig. S4**. **Predictive power of mRNA and protein expressions of proteome-specific DEPs** to predict patients’ survival in lung adenocarcinoma cohort in **Table S1.** A**. Z-score distributions to evaluate the expression differences between the deceased and alive patients at 12-months follow-up** (x-axis: mRNA or protein expressions; y-axis: Z-score from the logistic regression to quantify differential expression between the deceased and alive). **B, C.** mRNA (**B**) and protein (**C**) expressions of SYF2, stratified by deceased or alive patients at 12-months follow-up (in x-axis). **D. E.** mRNA (**D**) and protein (**E**) expressions of **MAN1A2**, stratified by deceased or alive patients at 12-months follow-up (in x-axis).


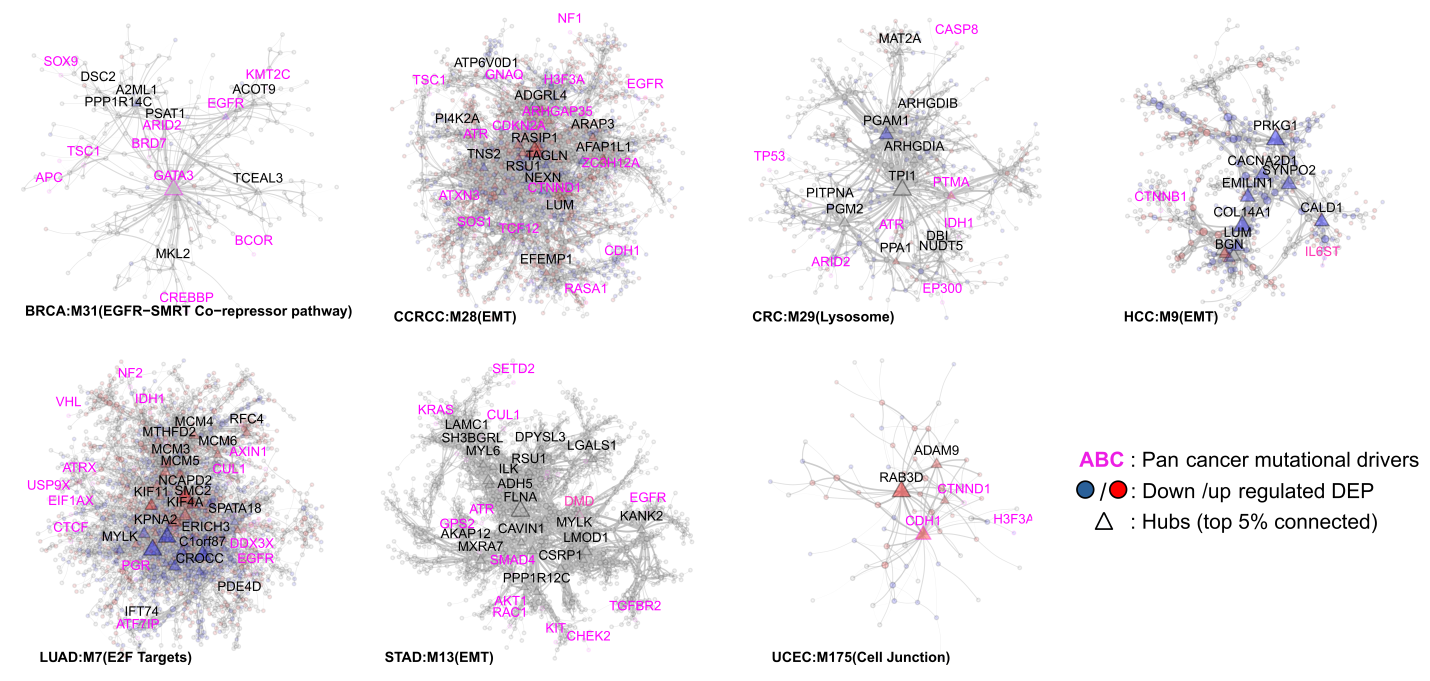


**Fig. S5**. **Subnetwork plots of top protein modules per cancer in** **Fig. 1E**. As shown on the bottom right, pan-cancer mutational drivers are labeled by magenta labels, down-/up-regulated DEPs in tumor are marked by blue/red colored nodes, and top 5% most connected hubs are shown as triangle nodes.


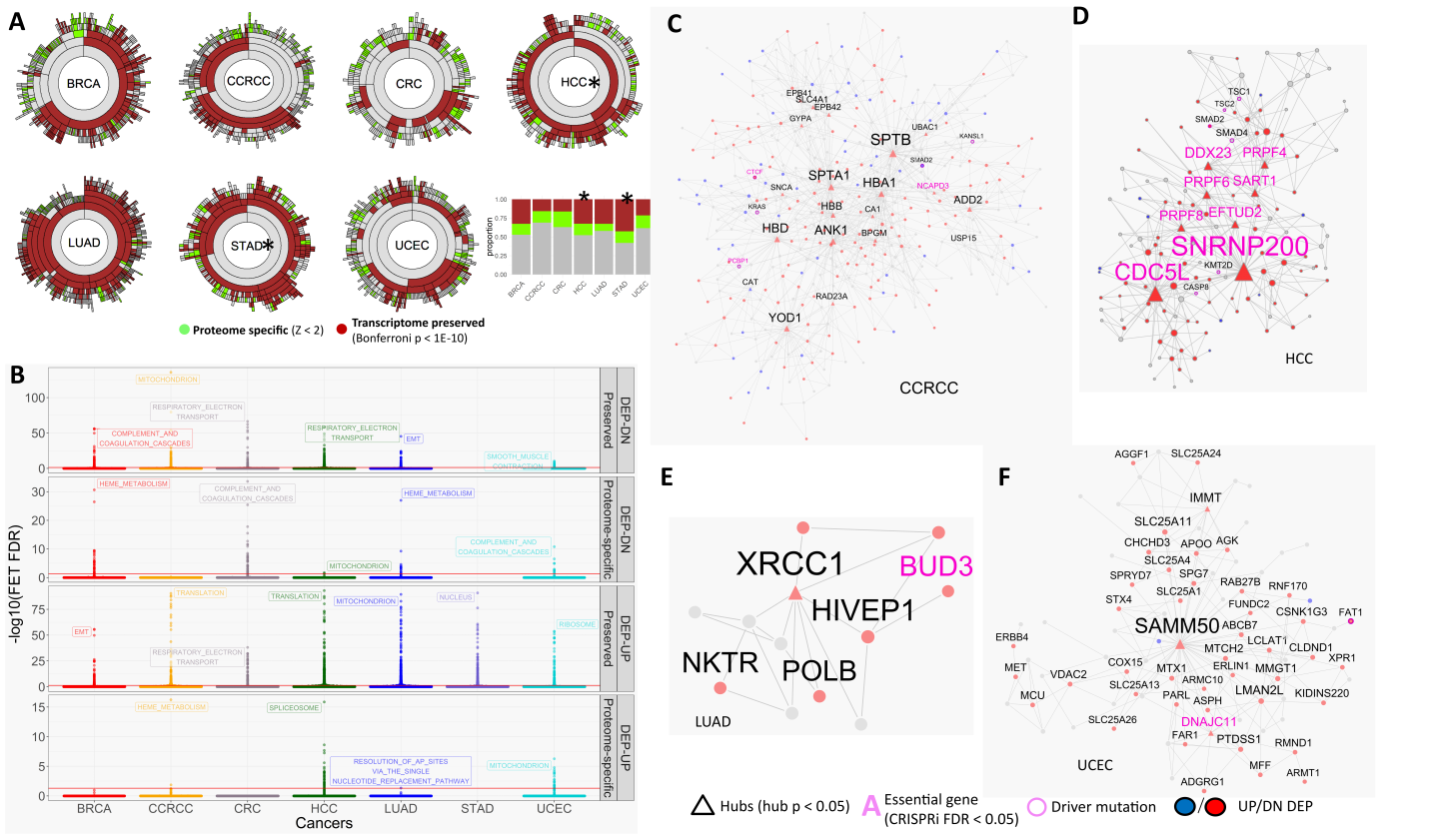


Fig. S6. Preserved or proteome-specific protein co-expressed modules in the respective transcriptome. A. The sunburst plots are organized according to the module hierarchy identified by MEGENA, and proteome-specific/transcriptome-preserved modules in both of PanCanAtlas and CPTAC are highlighted by green/brown. The barplot summarizes the overall results per cancer. B. Top represented pathways in proteome-specific or transcriptome preserved protein modules that are differentially expressed in the seven cancer proteomes. DEP signature enrichments and the module preservation calls (proteome-specific/conserved in transcriptome) are marked on the right. The y-axis –log10(FET FDR) for the enrichment of the respective pathways in the protein modules. The top two significant pathways in each case are labeled. C-F. Up-regulated proteome-specific modules enriched for the proteome-specific up-regulated pathways in D.


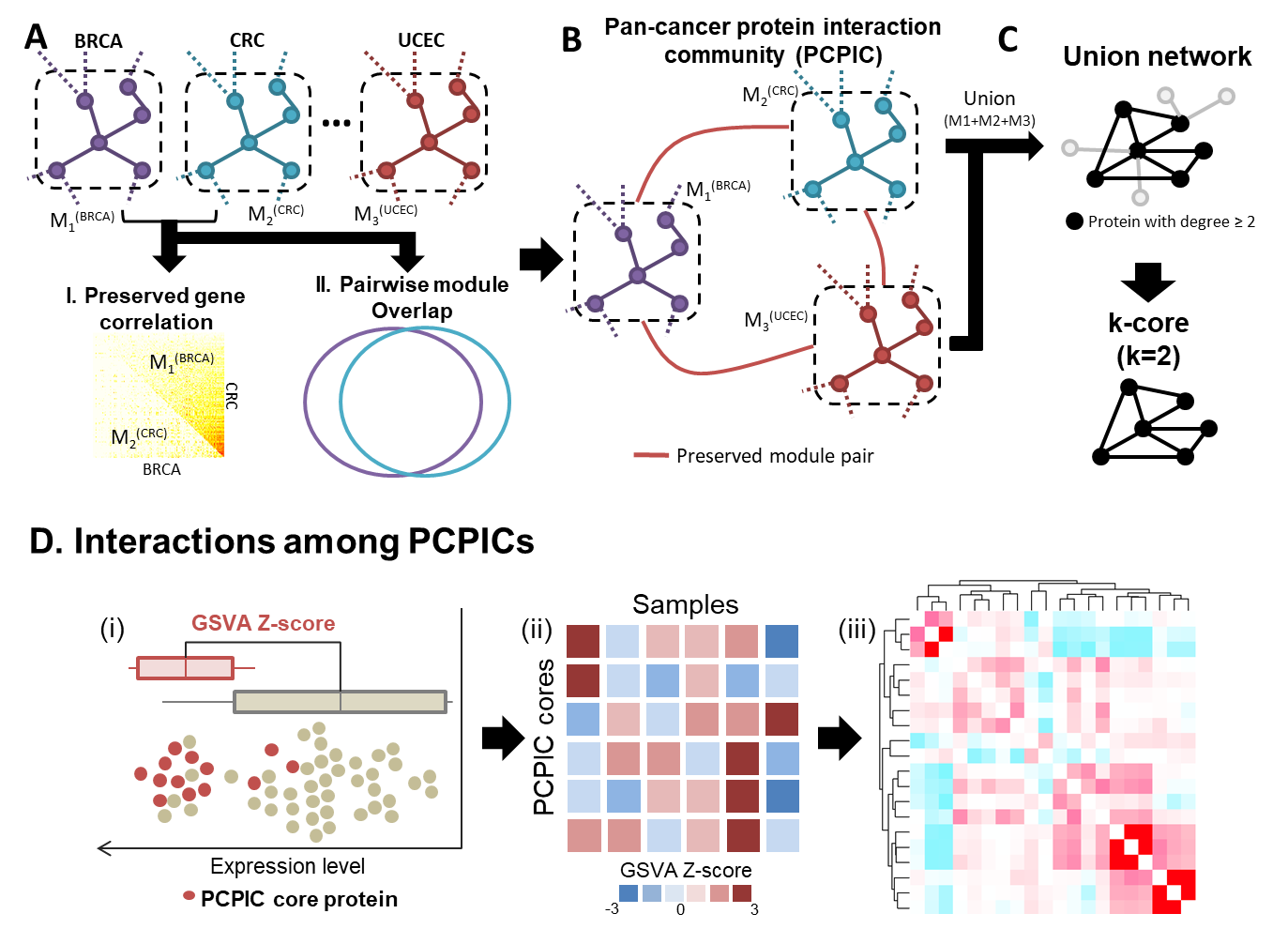


Fig. S7. Workflow description of pan-cancer protein interaction community (PCPIC) analysis. A. Identification of preserved modules across different cancer types. Top: A pair of co-expressed modules from different cancer types (in dashed rectangles) are compared. Here, we demonstrate an example of comparing a module from breast cancer (M_1_^(BRCA)^) with another from colorectal cancer (M_2_^(CRC)^). Bottom: Two criteria to call the module pair as cross-cancer preserved. *I. Module preservation:* Proteins in a breast cancer module, M1, should be significantly correlated in colorectal cancer proteome (Bonferroni adjusted module preservation p-value < 1E-10^5^). Similarly, a colorectal module, M2, should be significantly correlated in breast cancer proteome. *II. Module overlap:* M1 and M2 should show significant overlap with FDR adjusted Fisher’s Exact Test (FET) p-value < 0.05. B. Identification of pan-cancer protein interaction community (PCPIC): The pairs of preserved modules from A form a network of modules. By searching for groups of tightly linked modules in this network, a module of preserved modules (hence, Module-of-Modules) are identified. C. Identification of highly connected subnetwork as PIPIC core. Top: Union network by merging module subnetworks is formed. Bottom: A connected subnetwork formed by proteins with at least degree ≥ 2, hence 2-core, is identified identified as the PCPIC core. D. Workflow to evaluate inter-PCPIC interactions per cancer proteome. (i) *Calculation of GSVA Z-score per sample*: Per proteome sample, Gene Set Variation Analysis (GSVA) calculates z-score of a PCPIC core (marked red) as overall up-/down-regulation of the proteins, compared to the other protein expressions. (ii) *GSVA z-score matrix*: The rows are individual PCPIC cores, and columns individual samples. The heatmap shows different levels of regulations per PCPIC core. (iii) *Pairwise correlations between GSVA z-scores of PCPIC cores*: The interactions between two PCPIC cores are evaluated by Spearman’s correlation of respective GSVA z-scores.


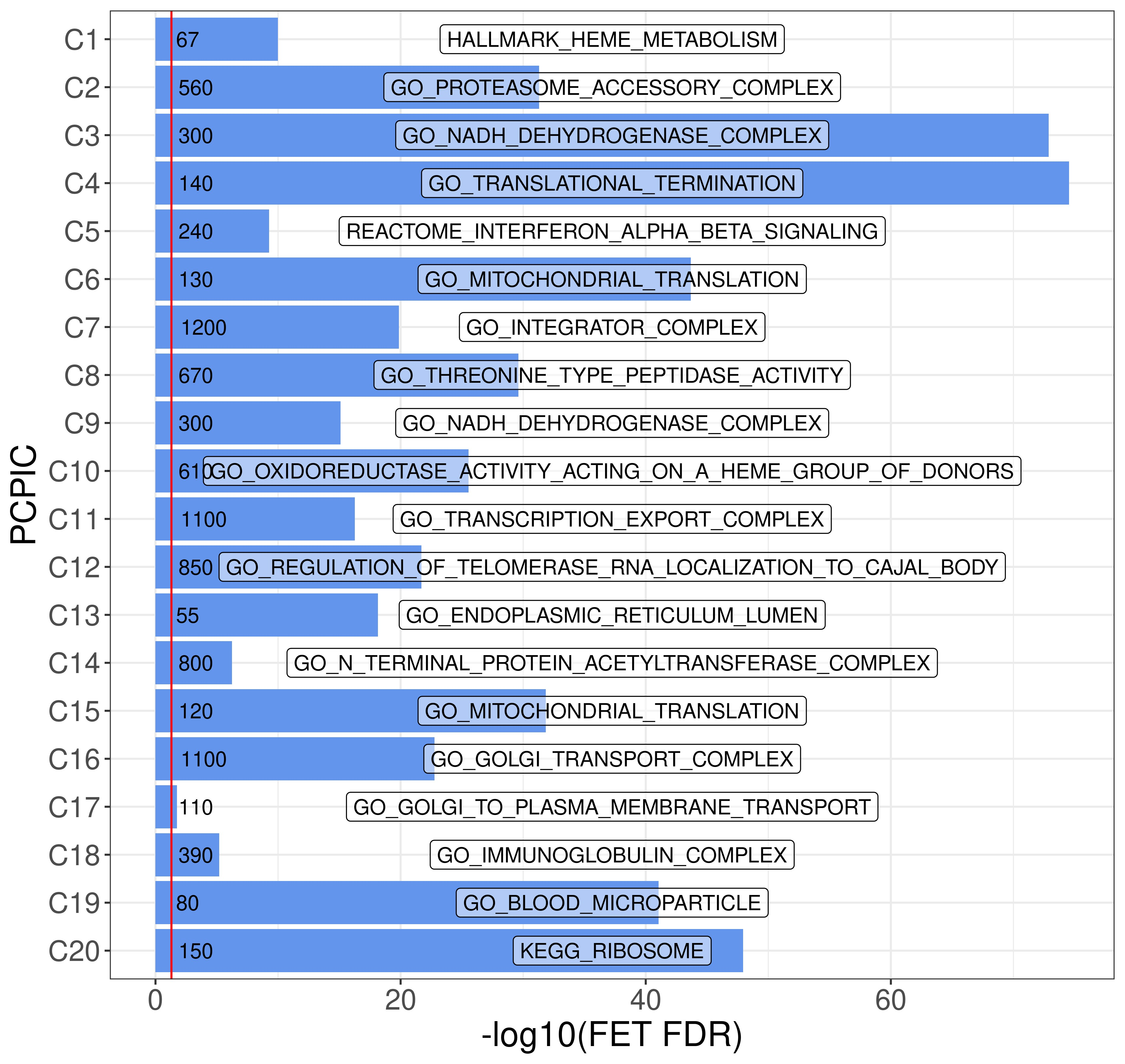


Fig. S8. Most enriched pathways core components of PCPICs. X-axis is –log10(FDR corrected FET p-value), y-axis contains core components of PCPICs. The numbers on the bars are enrichment fold changes and the written terms are the most enriched pathways.


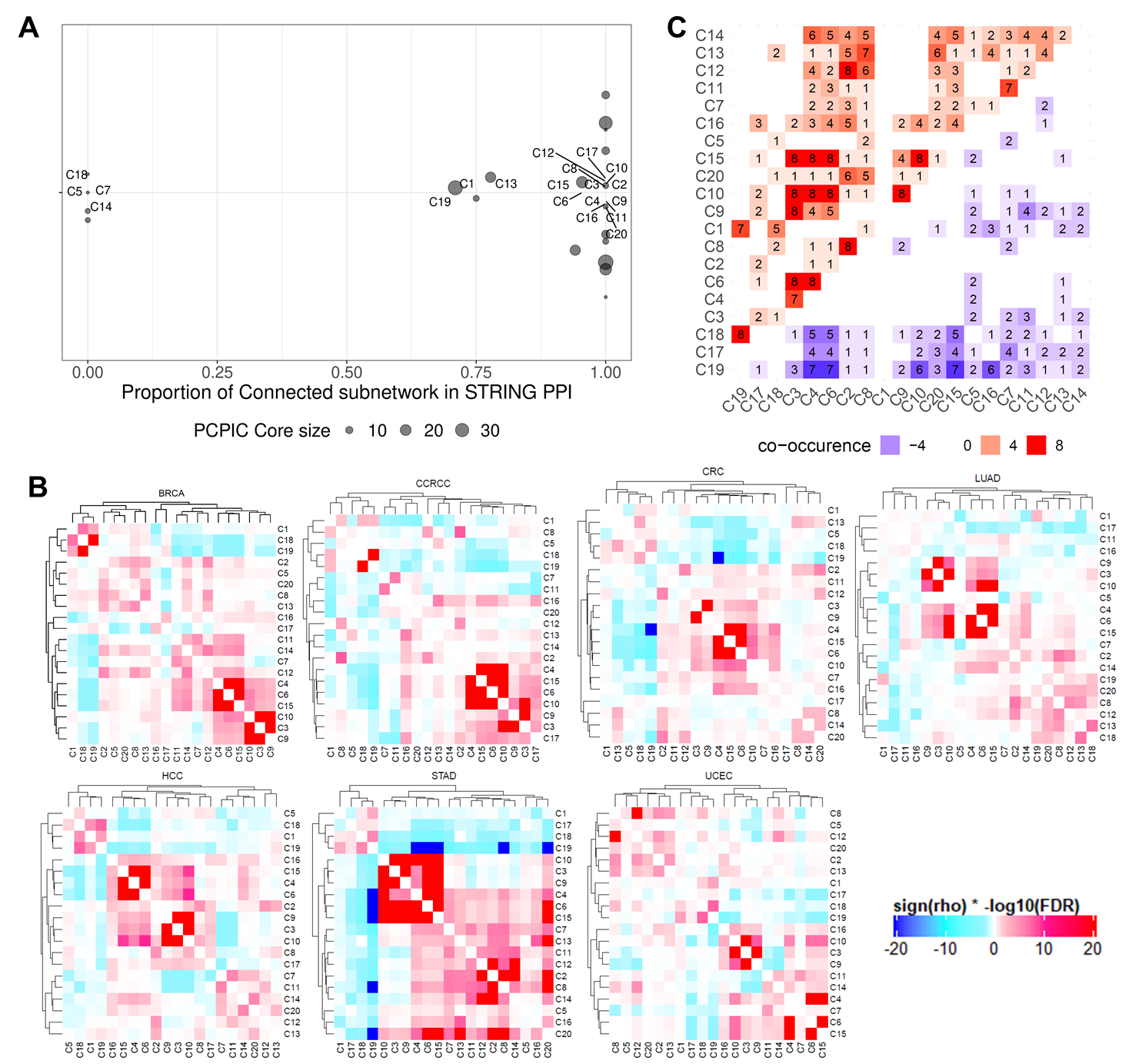


Fig. S9. Cross-talk across distinct PCPICs. A. X-axis is the proportion of connected subnetwork (i.e. giant component) by Protein-Protein Interactions (PPI) curated in STRING database (PPI confidence threshold = 700) for each PCPIC core. Proportion = 1 indicates the entire proteins in the PCPIC core are connected as a single network by known PPI links, and proportion = 0 means all proteins are disconnected. B. Correlation between GSVA scores of PCPIC cores in each cancer proteome. Red/blue color represents significant positive/negative correlation between the respective pair of PCPIC cores. C. Instances of correlated pairs of PCPIC cores across the seven cancers. Red or blue color represents a positive (upper triangular matrix) or negative (lower triangular matrix) correlation pair. The numbers in the heatmap are number of instances that the respective PCPIC core pair is significantly correlated with FDR < 0.05.


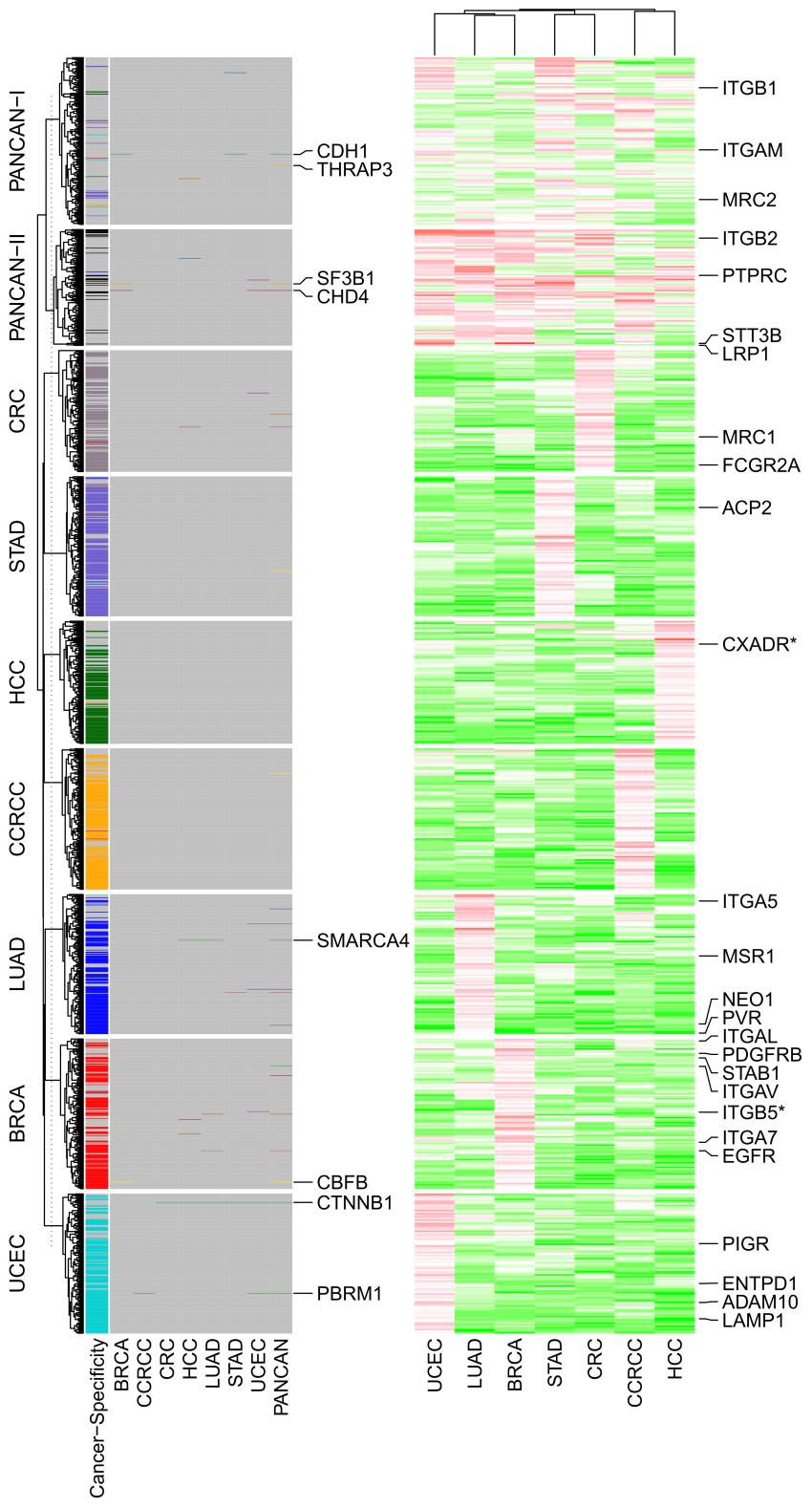


Fig. S10. Top hub genes in protein co-expression networks. Utilizing hub p-values from MEGENA, we identified hub genes from each cancer proteome network. Left annotation: Cancer type-specific hubs are marked by respective colors in legend below, where pan-cancer hubs (observed across at least 3 cancer types) are marked black. The mutational driver genes curated by TCGA pan-cancer study^119^ is highlighted in the middle. Driver mutations coinciding with hub genes are marked by respective gene symbols on the right. Right annotation: The heatmap depicts -log10(hub p-value) as identified by MEGENA in different cancer type networks. Hub genes identified as surface proteins, curated by mass-spectrometry derived Cell Surface Protein Atlas (CSPA)^120^, are marked by respective gene symbols.


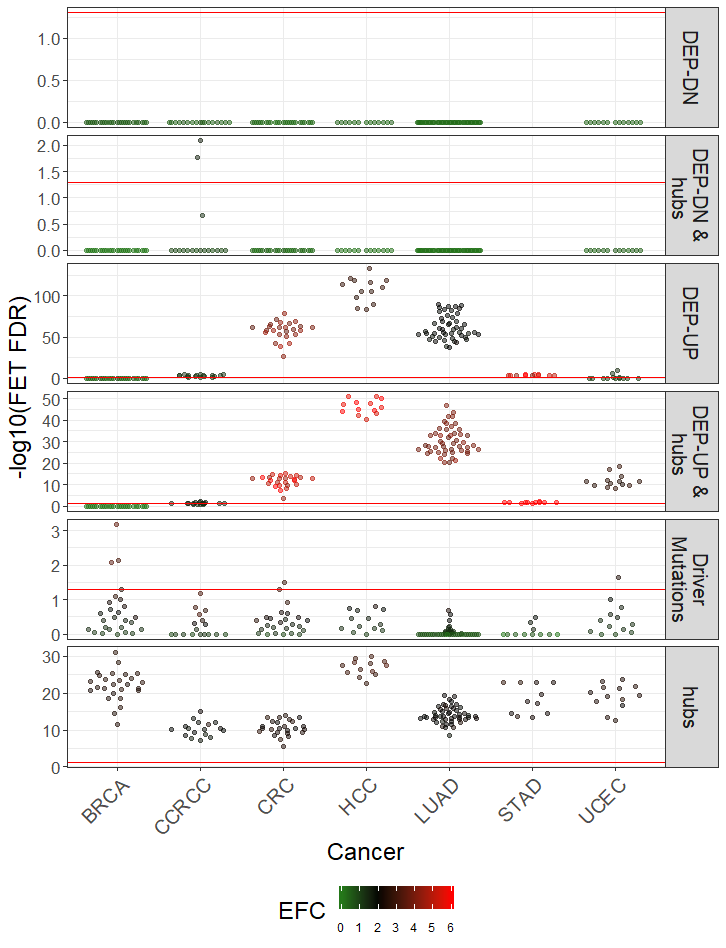


Fig. S11. Enrichments of essential genes in cancer cell line from Archilles database (FDR < 0.05) in various protein signatures. y-axis is –log10(FDR corrected FET p-value). Each dot represents a list of essential genes from a particular cancer cell line enriched in the respective protein signatures labeled on the right boxes. The enrichment fold change (i.e. ratio between actual overalp and expected overlap). The horizontal red line corresponds to FET FDR = 0.05 threshold.


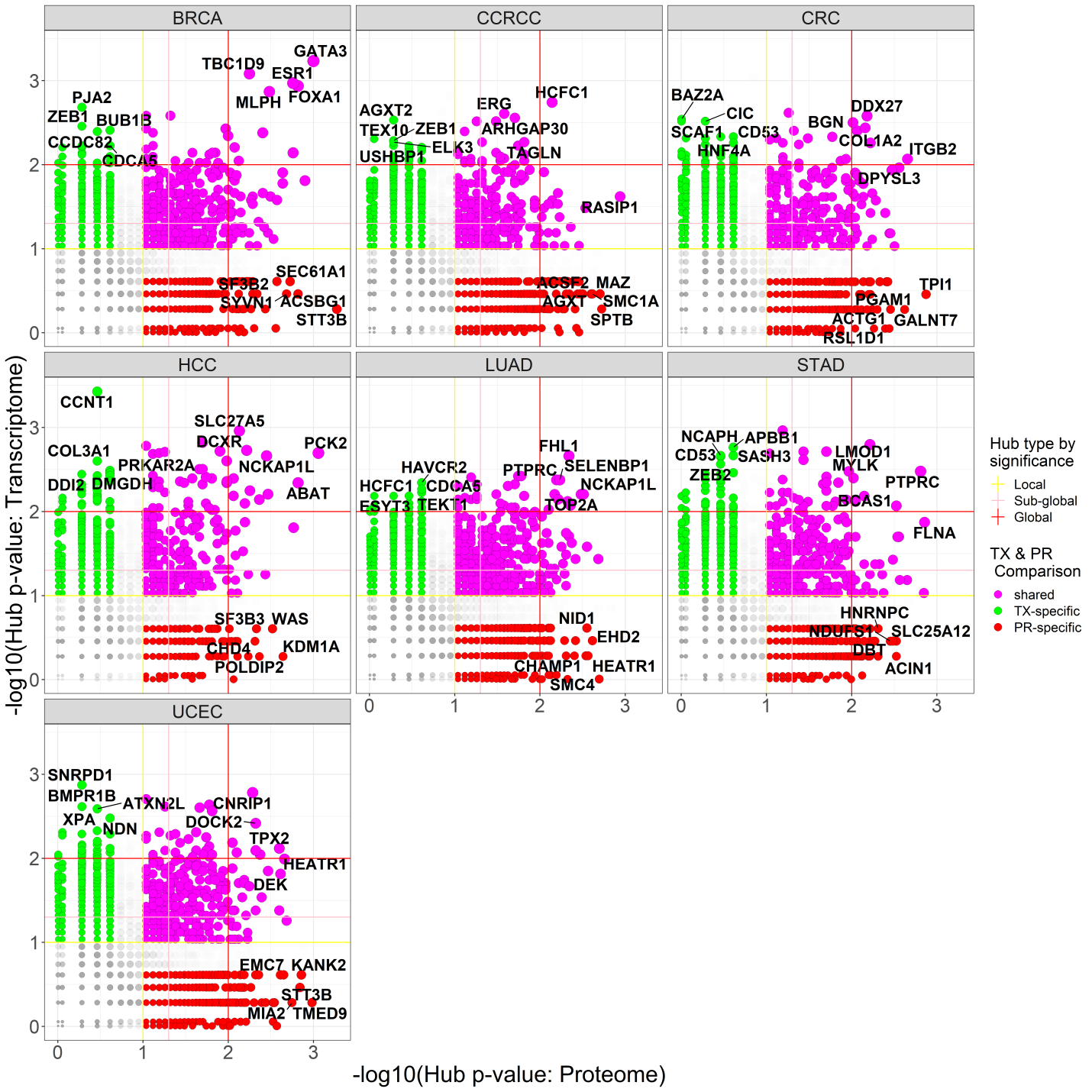


Fig. S12. Comparison of proteome (PR) and transcriptome (TX) network connectivities per cancer (x-axis: -log10(proteome hub p-value), y-axis: -log10(transcriptome hub p-value)). Proteome- or transcriptome-specific hubs or shared hubs in proteome and transcriptome are color-coded points as shown in the right-bottom legend. Various hub p-value thresholds to define local, sub-global and global hubs are drawn into color-coded lines as shown on the right-top legend.

**
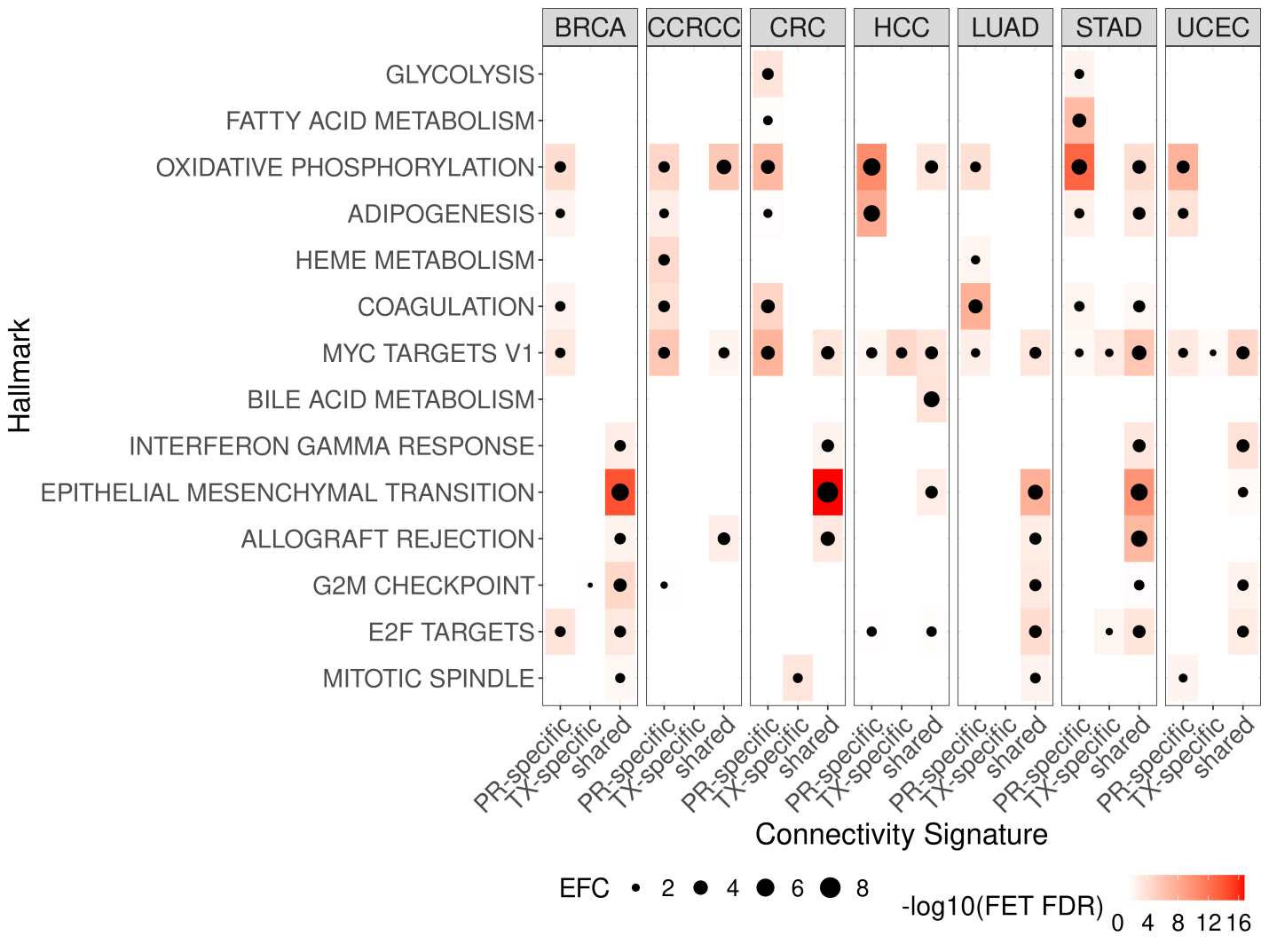
**

Fig. S13. Enriched hallmark pathways in proteome- (PR) or transcriptome-(TX) specific hub genes, or shared hub genes in PR and TX. The heatmap shows –log10(FDR corrected FET p-value) for enrichment of cancer type-wise hub gene signatures (PR-/TX-specific or shared) in the hallmark pathway signatures.


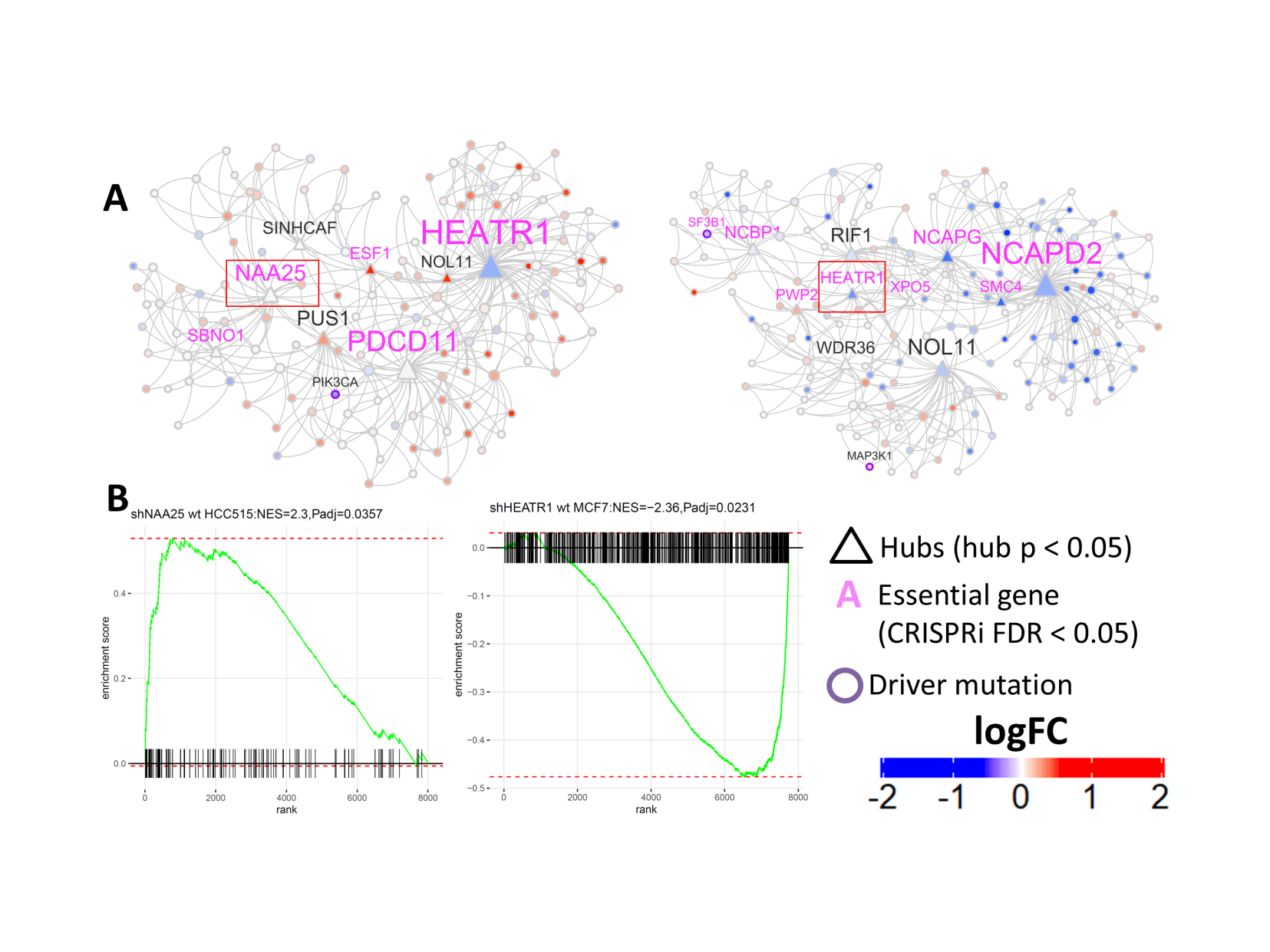


**Fig. S14**. **Validated drivers by gene perturbations signatures in cancer cells from LINCS database.** A. 2-layer neighborhoods of *NAA25* and *HEATR1* in the protein co-expression networks. The top 5% hubs in the neighborhood are labeled as triangles, essential lung cancer genes as purple gene symbols, driver mutations as purple borders, and log fold changes (logFC) greater than log2(1.2) or less than -log2(1.2) are highlighted by red or blue. Left is *NAA25* neighborhood network in LUAD, and right is *HEART1* neighborhood network in BRCA. B. Enrichments of gene perturbation signatures in the 2-layer neighborhoods of *NAA25* in LUAD (left) and *HEATR1* in BRCA (right).

**
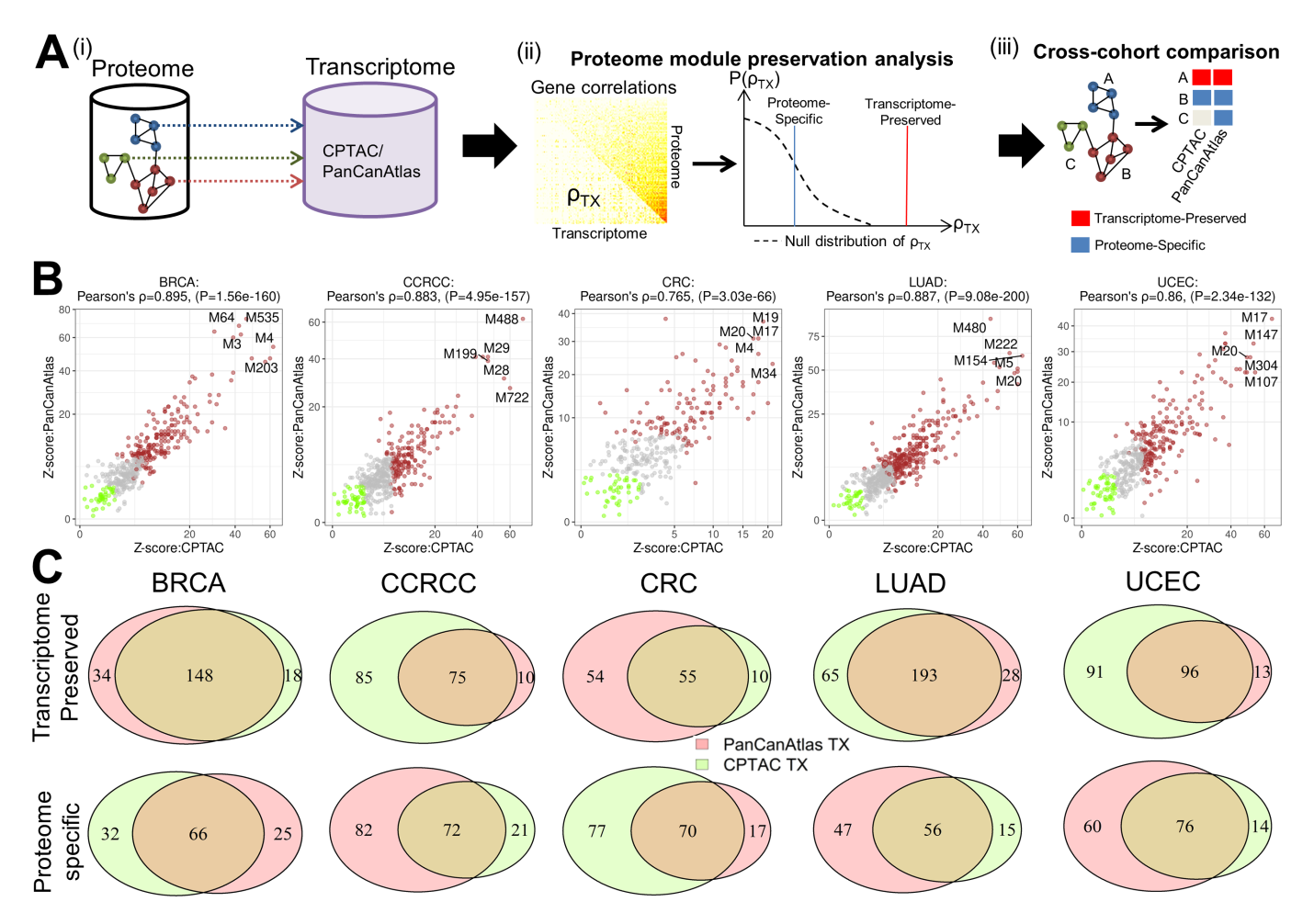
**

**Fig. S15**. **Evaluation of TCGA Pan-cancer atlas (PanCanAtlas) and CPTAC transcriptome (TX) cohorts for proteome module preservation analysis. A. Analysis workflow:** We designed comparative analysis workflow to compare the results from using PanCanAtlas and CPTAC transcriptome data. (i) Co-expressed proteome modules from each cancer type is evaluated for their degree of module preservations in the respective cancer transcriptome data from PanCanAtlas and CPTAC. (ii) For each proteome module, their gene correlations in the respective transcriptome data are evaluated by module preservation analysis (on left)^5^. The overall gene expression correlations (ρ_TX_) are compared to the null distributions from permuted data (on right). High ρ_TX_ that significantly deviates from the null distributions (colored in red horizontal line) with Bonferroni corrected p-value < 1E-10 is identified as transcriptome-preserved module. Low ρ_TX_ with Z-score < 2 (colored in blue horizontal line) are identified as no significant interactions in the transcriptome, hence proteome-specific module. (iii) Each proteome module is marked as transcriptome-preserved (red box), proteome-specific (blue box) or neither (grey box) when compared to transcriptome data from PanCanAtlas or CPTAC. The results from these two transcriptome cohorts are compared to justify the robustness of the proteome module preservation analysis results. **B. Scatter plot of proteome module preservation z-scores in CPTAC (x-axis) and PanCanAtlas (y-axis) transcriptomes**. Top 5 preserved proteome modules are labeled. Pearson’s correlation coefficient (ρ) and respective p-value for each cancer type is shown at the top. **C**. **Detection of proteome modules whose interactions were preserved in respective cancer transcriptome (top) or proteome-specific (bottom)**. Top: Venn diagrams show transcriptome-preserved proteome modules detected by comparing to PanCanAtlas (red) and CPTAC (green) transcriptomes. The overlap is highlighted in orange. Bottom: Proteome-specific modules detected by comparing their gene interactions in PanCanAtlas (red) and CPTAC (green) transcriptomes.

**REFERENCES**

1. Raab-Traub N. EBV-induced oncogenesis. In: Arvin A, Campadelli-Fiume G, Mocarski E, Moore PS, Roizman B, Whitley R, Yamanishi K, editors. Human Herpesviruses: Biology, Therapy, and Immunoprophylaxis. Cambridge2007.

2. Song W-M, Zhang B. Multiscale embedded gene co-expression network analysis. PLoS computational biology. 2015;11(11):e1004574.

3. Xu JY, Zhang C, Wang X, Zhai L, Ma Y, Mao Y, Qian K, Sun C, Liu Z, Jiang S, Wang M, Feng L, Zhao L, Liu P, Wang B, Zhao X, Xie H, Yang X, Zhao L, Chang Y, Jia J, Wang X, Zhang Y, Wang Y, Yang Y, Wu Z, Yang L, Liu B, Zhao T, Ren S, Sun A, Zhao Y, Ying W, Wang F, Wang G, Zhang Y, Cheng S, Qin J, Qian X, Wang Y, Li J, He F, Xiao T, Tan M. Integrative Proteomic Characterization of Human Lung Adenocarcinoma. Cell. 2020;182(1):245-61 e17. Epub 2020/07/11. doi: 10.1016/j.cell.2020.05.043. PubMed PMID: 32649877.

4. Takaku M, Grimm SA, Wade PA. GATA3 in Breast Cancer: Tumor Suppressor or Oncogene? Gene Expr. 2015;16(4):163-8. Epub 2015/12/08. doi: 10.3727/105221615X14399878166113. PubMed PMID: 26637396; PMCID: PMC4758516.

5. Miyazaki J, Ito K, Fujita T, Matsuzaki Y, Asano T, Hayakawa M, Asano T, Kawakami Y. Progression of Human Renal Cell Carcinoma via Inhibition of RhoA-ROCK Axis by PARG1. Transl Oncol. 2017;10(2):142-52. Epub 2017/01/31. doi: 10.1016/j.tranon.2016.12.004. PubMed PMID: 28131798; PMCID: PMC5284488.

6. Mygind KJ, Schwarz J, Sahgal P, Ivaska J, Kveiborg M. Loss of ADAM9 expression impairs beta1 integrin endocytosis, focal adhesion formation and cancer cell migration. J Cell Sci. 2018;131(1). Epub 2017/11/17. doi: 10.1242/jcs.205393. PubMed PMID: 29142101.

7. Koussounadis A, Langdon SP, Um IH, Harrison DJ, Smith VA. Relationship between differentially expressed mRNA and mRNA-protein correlations in a xenograft model system. Sci Rep. 2015;5:10775. Epub 2015/06/09. doi: 10.1038/srep10775. PubMed PMID: 26053859; PMCID: PMC4459080.

8. Schwanhausser B, Busse D, Li N, Dittmar G, Schuchhardt J, Wolf J, Chen W, Selbach M. Global quantification of mammalian gene expression control. Nature. 2011;473(7347):337-42. Epub 2011/05/20. doi: 10.1038/nature10098. PubMed PMID: 21593866.

9. Cao LL, Riascos-Bernal DF, Chinnasamy P, Dunaway CM, Hou R, Pujato MA, O'Rourke BP, Miskolci V, Guo L, Hodgson L, Fiser A, Sibinga NE. Control of mitochondrial function and cell growth by the atypical cadherin Fat1. Nature. 2016;539(7630):575-8. Epub 2016/11/10. doi: 10.1038/nature20170. PubMed PMID: 27828948; PMCID: PMC5257202.

10. Fares J, Fares MY, Khachfe HH, Salhab HA, Fares Y. Molecular principles of metastasis: a hallmark of cancer revisited. Signal Transduct Target Ther. 2020;5(1):28. Epub 2020/04/17. doi: 10.1038/s41392-020-0134-x. PubMed PMID: 32296047; PMCID: PMC7067809.

11. Hanahan D, Weinberg RA. Hallmarks of cancer: the next generation. Cell. 2011;144(5):646-74. Epub 2011/03/08. doi: 10.1016/j.cell.2011.02.013. PubMed PMID: 21376230.

12. Liberzon A, Birger C, Thorvaldsdottir H, Ghandi M, Mesirov JP, Tamayo P. The Molecular Signatures Database (MSigDB) hallmark gene set collection. Cell Syst. 2015;1(6):417-25. Epub 2016/01/16. doi: 10.1016/j.cels.2015.12.004. PubMed PMID: 26771021; PMCID: PMC4707969.

13. Szklarczyk D, Franceschini A, Wyder S, Forslund K, Heller D, Huerta-Cepas J, Simonovic M, Roth A, Santos A, Tsafou KP, Kuhn M, Bork P, Jensen LJ, von Mering C. STRING v10: protein-protein interaction networks, integrated over the tree of life. Nucleic Acids Res. 2015;43(Database issue):D447-52. Epub 2014/10/30. doi: 10.1093/nar/gku1003. PubMed PMID: 25352553; PMCID: PMC4383874.

14. Hanzelmann S, Castelo R, Guinney J. GSVA: gene set variation analysis for microarray and RNA-seq data. BMC Bioinformatics. 2013;14:7. Epub 2013/01/18. doi: 10.1186/1471-2105-14-7. PubMed PMID: 23323831; PMCID: PMC3618321.

15. Huang S, Wang Y. Golgi structure formation, function, and post-translational modifications in mammalian cells. F1000Res. 2017;6:2050. Epub 2017/12/12. doi: 10.12688/f1000research.11900.1. PubMed PMID: 29225785; PMCID: PMC5710388.

16. Aksnes H, Ree R, Arnesen T. Co-translational, Post-translational, and Non-catalytic Roles of N-Terminal Acetyltransferases. Mol Cell. 2019;73(6):1097-114. Epub 2019/03/18. doi: 10.1016/j.molcel.2019.02.007. PubMed PMID: 30878283; PMCID: PMC6962057.

17. Pelletier J, Thomas G, Volarevic S. Ribosome biogenesis in cancer: new players and therapeutic avenues. Nat Rev Cancer. 2018;18(1):51-63. Epub 2017/12/02. doi: 10.1038/nrc.2017.104. PubMed PMID: 29192214.

18. Lin TC, Su CY, Wu PY, Lai TC, Pan WA, Jan YH, Chang YC, Yeh CT, Chen CL, Ger LP, Chang HT, Yang CJ, Huang MS, Liu YP, Lin YF, Shyy JY, Tsai MD, Hsiao M. The nucleolar protein NIFK promotes cancer progression via CK1alpha/beta-catenin in metastasis and Ki-67-dependent cell proliferation. Elife. 2016;5. Epub 2016/03/18. doi: 10.7554/eLife.11288. PubMed PMID: 26984280; PMCID: PMC4811767.

19. Liu X, Gao Q, Li P, Zhao Q, Zhang J, Li J, Koseki H, Wong J. UHRF1 targets DNMT1 for DNA methylation through cooperative binding of hemi-methylated DNA and methylated H3K9. Nat Commun. 2013;4:1563. Epub 2013/03/07. doi: 10.1038/ncomms2562. PubMed PMID: 23463006.

20. Jiang H, Muir RK, Gonciarz RL, Olshen AB, Yeh I, Hann BC, Zhao N, Wang YH, Behr SC, Korkola JE, Evans MJ, Collisson EA, Renslo AR. Ferrous iron-activatable drug conjugate achieves potent MAPK blockade in KRAS-driven tumors. J Exp Med. 2022;219(4). Epub 2022/03/10. doi: 10.1084/jem.20210739. PubMed PMID: 35262628; PMCID: PMC8916116 reported grants from CTT, and personal fees from AAA Novartis and Genvivo outside the submitted work. J. Korkola reported "other" from Convergent Genomics outside the submitted work. M.J. Evans reported a patent to Trioxolane Agents (PCT/US2018/039768) pending. E.A. Collisson reported grants from Astra Zeneca and Bayer outside the submitted work. E.A. Collisson and A.R. Renslo are cofounders of Tatara and have an ownership interest in the company. A.R. Renslo reported personal fees from Tatara Therapeutics outside the submitted work; in addition, A.R. Renslo had a patent number 10,287,312 issued, a patent number 10,662,215 issued, a patent number 11,014,955 issued, and a patent number 11,072,594 issued. No other disclosures were reported.

21. Tripathi V, Sixt KM, Gao S, Xu X, Huang J, Weigert R, Zhou M, Zhang YE. Direct Regulation of Alternative Splicing by SMAD3 through PCBP1 Is Essential to the Tumor-Promoting Role of TGF-beta. Mol Cell. 2016;64(3):549-64. Epub 2016/10/18. doi: 10.1016/j.molcel.2016.09.013. PubMed PMID: 27746021; PMCID: PMC5123764.

22. Cornu M, Oppliger W, Albert V, Robitaille AM, Trapani F, Quagliata L, Fuhrer T, Sauer U, Terracciano L, Hall MN. Hepatic mTORC1 controls locomotor activity, body temperature, and lipid metabolism through FGF21. Proc Natl Acad Sci U S A. 2014;111(32):11592-9. Epub 2014/08/02. doi: 10.1073/pnas.1412047111. PubMed PMID: 25082895; PMCID: PMC4136616.

23. Brion C, Lutz SM, Albert FW. Simultaneous quantification of mRNA and protein in single cells reveals post-transcriptional effects of genetic variation. Elife. 2020;9. Epub 2020/11/17. doi: 10.7554/eLife.60645. PubMed PMID: 33191917; PMCID: PMC7707838.

24. Solaini G, Sgarbi G, Baracca A. Oxidative phosphorylation in cancer cells. Biochim Biophys Acta. 2011;1807(6):534-42. Epub 2010/09/21. doi: 10.1016/j.bbabio.2010.09.003. PubMed PMID: 20849810.

25. Santidrian AF, Matsuno-Yagi A, Ritland M, Seo BB, LeBoeuf SE, Gay LJ, Yagi T, Felding-Habermann B. Mitochondrial complex I activity and NAD+/NADH balance regulate breast cancer progression. J Clin Invest. 2013;123(3):1068-81. Epub 2013/02/22. doi: 10.1172/JCI64264. PubMed PMID: 23426180; PMCID: PMC3582128.

26. Li LD, Sun HF, Liu XX, Gao SP, Jiang HL, Hu X, Jin W. Down-Regulation of NDUFB9 Promotes Breast Cancer Cell Proliferation, Metastasis by Mediating Mitochondrial Metabolism. PLoS One. 2015;10(12):e0144441. Epub 2015/12/08. doi: 10.1371/journal.pone.0144441. PubMed PMID: 26641458; PMCID: PMC4671602.

27. Tsai YC, Weissman AM. The Unfolded Protein Response, Degradation from Endoplasmic Reticulum and Cancer. Genes Cancer. 2010;1(7):764-78. Epub 2011/02/19. doi: 10.1177/1947601910383011. PubMed PMID: 21331300; PMCID: PMC3039444.

28. Liu Y, Ye Y. Proteostasis regulation at the endoplasmic reticulum: a new perturbation site for targeted cancer therapy. Cell Res. 2011;21(6):867-83. Epub 2011/05/04. doi: 10.1038/cr.2011.75. PubMed PMID: 21537343; PMCID: PMC3203708.

29. Li X, Zhu F, Jiang J, Sun C, Zhong Q, Shen M, Wang X, Tian R, Shi C, Xu M, Peng F, Guo X, Hu J, Ye D, Wang M, Qin R. Simultaneous inhibition of the ubiquitin-proteasome system and autophagy enhances apoptosis induced by ER stress aggravators in human pancreatic cancer cells. Autophagy. 2016;12(9):1521-37. Epub 2016/06/17. doi: 10.1080/15548627.2016.1191722. PubMed PMID: 27308733; PMCID: PMC5082778.

30. Arnesen T, Starheim KK, Van Damme P, Evjenth R, Dinh H, Betts MJ, Ryningen A, Vandekerckhove J, Gevaert K, Anderson D. The chaperone-like protein HYPK acts together with NatA in cotranslational N-terminal acetylation and prevention of Huntingtin aggregation. Mol Cell Biol. 2010;30(8):1898-909. Epub 2010/02/16. doi: 10.1128/MCB.01199-09. PubMed PMID: 20154145; PMCID: PMC2849469.

31. Gottlieb L, Marmorstein R. Structure of Human NatA and Its Regulation by the Huntingtin Interacting Protein HYPK. Structure. 2018;26(7):925-35 e8. Epub 2018/05/15. doi: 10.1016/j.str.2018.04.003. PubMed PMID: 29754825; PMCID: PMC6031454.

32. Deng M, Li F, Ballif BA, Li S, Chen X, Guo L, Ye X. Identification and functional analysis of a novel cyclin e/cdk2 substrate ankrd17. J Biol Chem. 2009;284(12):7875-88. Epub 2009/01/20. doi: 10.1074/jbc.M807827200. PubMed PMID: 19150984; PMCID: PMC2658080.

33. Wang Y, Tong X, Li G, Li J, Deng M, Ye X. Ankrd17 positively regulates RIG-I-like receptor (RLR)-mediated immune signaling. Eur J Immunol. 2012;42(5):1304-15. Epub 2012/02/14. doi: 10.1002/eji.201142125. PubMed PMID: 22328336.

34. Menning M, Kufer TA. A role for the Ankyrin repeat containing protein Ankrd17 in Nod1- and Nod2-mediated inflammatory responses. FEBS Lett. 2013;587(14):2137-42. Epub 2013/05/29. doi: 10.1016/j.febslet.2013.05.037. PubMed PMID: 23711367.

35. Kalvik TV, Arnesen T. Protein N-terminal acetyltransferases in cancer. Oncogene. 2013;32(3):269-76. Epub 2012/03/07. doi: 10.1038/onc.2012.82. PubMed PMID: 22391571.

36. Reddi R, Saddanapu V, Chinthapalli DK, Sankoju P, Sripadi P, Addlagatta A. Human Naa50 Protein Displays Broad Substrate Specificity for Amino-terminal Acetylation: DETAILED STRUCTURAL AND BIOCHEMICAL ANALYSIS USING TETRAPEPTIDE LIBRARY. J Biol Chem. 2016;291(39):20530-8. Epub 2016/08/04. doi: 10.1074/jbc.M116.730432. PubMed PMID: 27484799; PMCID: PMC5034047.

37. Caesar R, Warringer J, Blomberg A. Physiological importance and identification of novel targets for the N-terminal acetyltransferase NatB. Eukaryot Cell. 2006;5(2):368-78. Epub 2006/02/10. doi: 10.1128/EC.5.2.368-378.2006. PubMed PMID: 16467477; PMCID: PMC1405896.

38. Eiyama A, Okamoto K. Protein N-terminal Acetylation by the NatA Complex Is Critical for Selective Mitochondrial Degradation. J Biol Chem. 2015;290(41):25034-44. Epub 2015/08/25. doi: 10.1074/jbc.M115.677468. PubMed PMID: 26296886; PMCID: PMC4599008.

39. van Deventer S, Menendez-Benito V, van Leeuwen F, Neefjes J. N-terminal acetylation and replicative age affect proteasome localization and cell fitness during aging. J Cell Sci. 2015;128(1):109-17. Epub 2014/11/22. doi: 10.1242/jcs.157354. PubMed PMID: 25413350; PMCID: PMC4282048.

40. Wang HZ, Yang SH, Li GY, Cao X. Subunits of human condensins are potential therapeutic targets for cancers. Cell Div. 2018;13:2. Epub 2018/02/23. doi: 10.1186/s13008-018-0035-3. PubMed PMID: 29467813; PMCID: PMC5819170.

41. Ham MF, Takakuwa T, Rahadiani N, Tresnasari K, Nakajima H, Aozasa K. Condensin mutations and abnormal chromosomal structures in pyothorax-associated lymphoma. Cancer Sci. 2007;98(7):1041-7. Epub 2007/05/10. doi: 10.1111/j.1349-7006.2007.00500.x. PubMed PMID: 17488335.

42. Murakami-Tonami Y, Kishida S, Takeuchi I, Katou Y, Maris JM, Ichikawa H, Kondo Y, Sekido Y, Shirahige K, Murakami H, Kadomatsu K. Inactivation of SMC2 shows a synergistic lethal response in MYCN-amplified neuroblastoma cells. Cell Cycle. 2014;13(7):1115-31. Epub 2014/02/21. doi: 10.4161/cc.27983. PubMed PMID: 24553121; PMCID: PMC4013162.

43. Je EM, Yoo NJ, Lee SH. Mutational and expressional analysis of SMC2 gene in gastric and colorectal cancers with microsatellite instability. APMIS. 2014;122(6):499-504. Epub 2014/02/04. doi: 10.1111/apm.12193. PubMed PMID: 24483990.

44. Wood JL, Liang Y, Li K, Chen J. Microcephalin/MCPH1 associates with the Condensin II complex to function in homologous recombination repair. J Biol Chem. 2008;283(43):29586-92. Epub 2008/08/23. doi: 10.1074/jbc.M804080200. PubMed PMID: 18718915; PMCID: PMC2570891.

45. Hervouet E, Peixoto P, Delage-Mourroux R, Boyer-Guittaut M, Cartron PF. Specific or not specific recruitment of DNMTs for DNA methylation, an epigenetic dilemma. Clin Epigenetics. 2018;10:17. Epub 2018/02/17. doi: 10.1186/s13148-018-0450-y. PubMed PMID: 29449903; PMCID: PMC5807744.

46. Hopfner R, Mousli M, Jeltsch JM, Voulgaris A, Lutz Y, Marin C, Bellocq JP, Oudet P, Bronner C. ICBP90, a novel human CCAAT binding protein, involved in the regulation of topoisomerase IIalpha expression. Cancer Res. 2000;60(1):121-8. Epub 2000/01/26. PubMed PMID: 10646863.

47. Ono T, Sakamoto C, Nakao M, Saitoh N, Hirano T. Condensin II plays an essential role in reversible assembly of mitotic chromosomes in situ. Mol Biol Cell. 2017;28(21):2875-86. Epub 2017/08/25. doi: 10.1091/mbc.E17-04-0252. PubMed PMID: 28835373; PMCID: PMC5638589.

48. Chang CJ, Hung MC. The role of EZH2 in tumour progression. Br J Cancer. 2012;106(2):243-7. Epub 2011/12/22. doi: 10.1038/bjc.2011.551. PubMed PMID: 22187039; PMCID: PMC3261672.

49. Kim E, Kerssemakers J, Shaltiel IA, Haering CH, Dekker C. DNA-loop extruding condensin complexes can traverse one another. Nature. 2020;579(7799):438-42. Epub 2020/03/07. doi: 10.1038/s41586-020-2067-5. PubMed PMID: 32132705.

50. Lin X, Leicher R, Liu S, Zhang B. Cooperative DNA looping by PRC2 complexes. Nucleic Acids Res. 2021;49(11):6238-48. Epub 2021/06/01. doi: 10.1093/nar/gkab441. PubMed PMID: 34057467; PMCID: PMC8216278.

51. Cheng Q, Yuan F, Lu F, Zhang B, Chen T, Chen X, Cheng Y, Li N, Ma L, Tong T. CSIG promotes hepatocellular carcinoma proliferation by activating c-MYC expression. Oncotarget. 2015;6(7):4733-44. Epub 2015/03/10. doi: 10.18632/oncotarget.2900. PubMed PMID: 25749381; PMCID: PMC4467111.

52. Li XP, Jiao JU, Lu LI, Zou Q, Zhu S, Zhang Y. Overexpression of ribosomal L1 domain containing 1 is associated with an aggressive phenotype and a poor prognosis in patients with prostate cancer. Oncol Lett. 2016;11(4):2839-44. Epub 2016/04/14. doi: 10.3892/ol.2016.4294. PubMed PMID: 27073561; PMCID: PMC4812176.

53. Ma L, Chang N, Guo S, Li Q, Zhang Z, Wang W, Tong T. CSIG inhibits PTEN translation in replicative senescence. Mol Cell Biol. 2008;28(20):6290-301. Epub 2008/08/06. doi: 10.1128/MCB.00142-08. PubMed PMID: 18678645; PMCID: PMC2577433.

54. Calo E, Flynn RA, Martin L, Spitale RC, Chang HY, Wysocka J. RNA helicase DDX21 coordinates transcription and ribosomal RNA processing. Nature. 2015;518(7538):249-53. Epub 2014/12/04. doi: 10.1038/nature13923. PubMed PMID: 25470060; PMCID: PMC4827702.

55. Song C, Hotz-Wagenblatt A, Voit R, Grummt I. SIRT7 and the DEAD-box helicase DDX21 cooperate to resolve genomic R loops and safeguard genome stability. Genes Dev. 2017;31(13):1370-81. Epub 2017/08/10. doi: 10.1101/gad.300624.117. PubMed PMID: 28790157; PMCID: PMC5580657.

56. Fuller-Pace FV. DEAD box RNA helicase functions in cancer. RNA Biol. 2013;10(1):121-32. Epub 2013/01/29. doi: 10.4161/rna.23312. PubMed PMID: 23353573; PMCID: PMC3590229.

57. Behan FM, Iorio F, Picco G, Goncalves E, Beaver CM, Migliardi G, Santos R, Rao Y, Sassi F, Pinnelli M, Ansari R, Harper S, Jackson DA, McRae R, Pooley R, Wilkinson P, van der Meer D, Dow D, Buser-Doepner C, Bertotti A, Trusolino L, Stronach EA, Saez-Rodriguez J, Yusa K, Garnett MJ. Prioritization of cancer therapeutic targets using CRISPR-Cas9 screens. Nature. 2019;568(7753):511-6. Epub 2019/04/12. doi: 10.1038/s41586-019-1103-9. PubMed PMID: 30971826.

58. Dong MB, Wang G, Chow RD, Ye L, Zhu L, Dai X, Park JJ, Kim HR, Errami Y, Guzman CD, Zhou X, Chen KY, Renauer PA, Du Y, Shen J, Lam SZ, Zhou JJ, Lannin DR, Herbst RS, Chen S. Systematic Immunotherapy Target Discovery Using Genome-Scale In Vivo CRISPR Screens in CD8 T Cells. Cell. 2019;178(5):1189-204 e23. Epub 2019/08/24. doi: 10.1016/j.cell.2019.07.044. PubMed PMID: 31442407; PMCID: PMC6719679.

59. He S, Ma X, Ye Y, Zhang M, Zhuang J, Song Y, Xia W. HEATR1 modulates cell survival in non-small cell lung cancer via activation of the p53/PUMA signaling pathway. Onco Targets Ther. 2019;12:4001-11. Epub 2019/06/14. doi: 10.2147/OTT.S195826. PubMed PMID: 31190896; PMCID: PMC6535672.

60. Wu ZB, Qiu C, Zhang AL, Cai L, Lin SJ, Yao Y, Tang QS, Xu M, Hua W, Chu YW, Mao Y, Zhu JH, Xu J, Zhou LF. Glioma-associated antigen HEATR1 induces functional cytotoxic T lymphocytes in patients with glioma. J Immunol Res. 2014;2014:131494. Epub 2014/08/16. doi: 10.1155/2014/131494. PubMed PMID: 25126583; PMCID: PMC4121097.

61. Collins NB, Abosy RA, Miller B, Bi K, Manguso R, Yates K, Haining WN. PI3K activated tumors evade tumor immunity by promoting an inhibitory myeloid microenvironment. The Journal of Immunology. 2019;202:58.17-58.17.

62. Borcoman E, De La Rochere P, Richer W, Vacher S, Chemlali W, Krucker C, Sirab N, Radvanyi F, Allory Y, Pignot G, Barry de Longchamps N, Damotte D, Meseure D, Sedlik C, Bieche I, Piaggio E. Inhibition of PI3K pathway increases immune infiltrate in muscle-invasive bladder cancer. Oncoimmunology. 2019;8(5):e1581556. Epub 2019/05/10. doi: 10.1080/2162402X.2019.1581556. PubMed PMID: 31069145; PMCID: PMC6492984.

63. Okkenhaug K, Graupera M, Vanhaesebroeck B. Targeting PI3K in Cancer: Impact on Tumor Cells, Their Protective Stroma, Angiogenesis, and Immunotherapy. Cancer Discov. 2016;6(10):1090-105. Epub 2016/09/23. doi: 10.1158/2159-8290.CD-16-0716. PubMed PMID: 27655435; PMCID: PMC5293166.

64. (CPTAC) CPTAC. Available from: https://cptac-data-portal.georgetown.edu/cptac/public?scope=Phase+III.

65. Vasaikar S, Huang C, Wang X, Petyuk VA, Savage SR, Wen B, Dou Y, Zhang Y, Shi Z, Arshad OA, Gritsenko MA, Zimmerman LJ, McDermott JE, Clauss TR, Moore RJ, Zhao R, Monroe ME, Wang YT, Chambers MC, Slebos RJC, Lau KS, Mo Q, Ding L, Ellis M, Thiagarajan M, Kinsinger CR, Rodriguez H, Smith RD, Rodland KD, Liebler DC, Liu T, Zhang B, Clinical Proteomic Tumor Analysis C. Proteogenomic Analysis of Human Colon Cancer Reveals New Therapeutic Opportunities. Cell. 2019;177(4):1035-49 e19. Epub 2019/04/30. doi: 10.1016/j.cell.2019.03.030. PubMed PMID: 31031003; PMCID: PMC6768830.

66. Jiang Y, Sun A, Zhao Y, Ying W, Sun H, Yang X, Xing B, Sun W, Ren L, Hu B, Li C, Zhang L, Qin G, Zhang M, Chen N, Zhang M, Huang Y, Zhou J, Zhao Y, Liu M, Zhu X, Qiu Y, Sun Y, Huang C, Yan M, Wang M, Liu W, Tian F, Xu H, Zhou J, Wu Z, Shi T, Zhu W, Qin J, Xie L, Fan J, Qian X, He F, Chinese Human Proteome Project C. Proteomics identifies new therapeutic targets of early-stage hepatocellular carcinoma. Nature. 2019;567(7747):257-61. Epub 2019/03/01. doi: 10.1038/s41586-019-0987-8. PubMed PMID: 30814741.

67. Mun DG, Bhin J, Kim S, Kim H, Jung JH, Jung Y, Jang YE, Park JM, Kim H, Jung Y, Lee H, Bae J, Back S, Kim SJ, Kim J, Park H, Li H, Hwang KB, Park YS, Yook JH, Kim BS, Kwon SY, Ryu SW, Park DY, Jeon TY, Kim DH, Lee JH, Han SU, Song KS, Park D, Park JW, Rodriguez H, Kim J, Lee H, Kim KP, Yang EG, Kim HK, Paek E, Lee S, Lee SW, Hwang D. Proteogenomic Characterization of Human Early-Onset Gastric Cancer. Cancer Cell. 2019;35(1):111-24 e10. Epub 2019/01/16. doi: 10.1016/j.ccell.2018.12.003. PubMed PMID: 30645970.

68. Langfelder P, Luo R, Oldham MC, Horvath S. Is my network module preserved and reproducible? PLoS Comput Biol. 2011;7(1):e1001057. Epub 2011/02/02. doi: 10.1371/journal.pcbi.1001057. PubMed PMID: 21283776; PMCID: PMC3024255.

69. Ritchie ME, Phipson B, Wu D, Hu Y, Law CW, Shi W, Smyth GK. limma powers differential expression analyses for RNA-sequencing and microarray studies. Nucleic Acids Res. 2015;43(7):e47. Epub 2015/01/22. doi: 10.1093/nar/gkv007. PubMed PMID: 25605792; PMCID: PMC4402510.

70. Clark DJ, Dhanasekaran SM, Petralia F, Pan J, Song X, Hu Y, da Veiga Leprevost F, Reva B, Lih TM, Chang HY, Ma W, Huang C, Ricketts CJ, Chen L, Krek A, Li Y, Rykunov D, Li QK, Chen LS, Ozbek U, Vasaikar S, Wu Y, Yoo S, Chowdhury S, Wyczalkowski MA, Ji J, Schnaubelt M, Kong A, Sethuraman S, Avtonomov DM, Ao M, Colaprico A, Cao S, Cho KC, Kalayci S, Ma S, Liu W, Ruggles K, Calinawan A, Gumus ZH, Geiszler D, Kawaler E, Teo GC, Wen B, Zhang Y, Keegan S, Li K, Chen F, Edwards N, Pierorazio PM, Chen XS, Pavlovich CP, Hakimi AA, Brominski G, Hsieh JJ, Antczak A, Omelchenko T, Lubinski J, Wiznerowicz M, Linehan WM, Kinsinger CR, Thiagarajan M, Boja ES, Mesri M, Hiltke T, Robles AI, Rodriguez H, Qian J, Fenyo D, Zhang B, Ding L, Schadt E, Chinnaiyan AM, Zhang Z, Omenn GS, Cieslik M, Chan DW, Nesvizhskii AI, Wang P, Zhang H, Clinical Proteomic Tumor Analysis C. Integrated Proteogenomic Characterization of Clear Cell Renal Cell Carcinoma. Cell. 2019;179(4):964-83 e31. Epub 2019/11/02. doi: 10.1016/j.cell.2019.10.007. PubMed PMID: 31675502; PMCID: PMC7331093.

71. Dou Y, Kawaler EA, Cui Zhou D, Gritsenko MA, Huang C, Blumenberg L, Karpova A, Petyuk VA, Savage SR, Satpathy S, Liu W, Wu Y, Tsai CF, Wen B, Li Z, Cao S, Moon J, Shi Z, Cornwell M, Wyczalkowski MA, Chu RK, Vasaikar S, Zhou H, Gao Q, Moore RJ, Li K, Sethuraman S, Monroe ME, Zhao R, Heiman D, Krug K, Clauser K, Kothadia R, Maruvka Y, Pico AR, Oliphant AE, Hoskins EL, Pugh SL, Beecroft SJI, Adams DW, Jarman JC, Kong A, Chang HY, Reva B, Liao Y, Rykunov D, Colaprico A, Chen XS, Czekanski A, Jedryka M, Matkowski R, Wiznerowicz M, Hiltke T, Boja E, Kinsinger CR, Mesri M, Robles AI, Rodriguez H, Mutch D, Fuh K, Ellis MJ, DeLair D, Thiagarajan M, Mani DR, Getz G, Noble M, Nesvizhskii AI, Wang P, Anderson ML, Levine DA, Smith RD, Payne SH, Ruggles KV, Rodland KD, Ding L, Zhang B, Liu T, Fenyo D, Clinical Proteomic Tumor Analysis C. Proteogenomic Characterization of Endometrial Carcinoma. Cell. 2020;180(4):729-48 e26. Epub 2020/02/16. doi: 10.1016/j.cell.2020.01.026. PubMed PMID: 32059776; PMCID: PMC7233456.

72. Ellis MJ, Gillette M, Carr SA, Paulovich AG, Smith RD, Rodland KK, Townsend RR, Kinsinger C, Mesri M, Rodriguez H, Liebler DC, Clinical Proteomic Tumor Analysis C. Connecting genomic alterations to cancer biology with proteomics: the NCI Clinical Proteomic Tumor Analysis Consortium. Cancer Discov. 2013;3(10):1108-12. Epub 2013/10/15. doi: 10.1158/2159-8290.CD-13-0219. PubMed PMID: 24124232; PMCID: PMC3800055.

73. Gao Q, Zhu H, Dong L, Shi W, Chen R, Song Z, Huang C, Li J, Dong X, Zhou Y, Liu Q, Ma L, Wang X, Zhou J, Liu Y, Boja E, Robles AI, Ma W, Wang P, Li Y, Ding L, Wen B, Zhang B, Rodriguez H, Gao D, Zhou H, Fan J. Integrated Proteogenomic Characterization of HBV-Related Hepatocellular Carcinoma. Cell. 2019;179(2):561-77 e22. Epub 2019/10/05. doi: 10.1016/j.cell.2019.08.052. PubMed PMID: 31585088.

74. Miller JA, Cai C, Langfelder P, Geschwind DH, Kurian SM, Salomon DR, Horvath S. Strategies for aggregating gene expression data: the collapseRows R function. BMC Bioinformatics. 2011;12:322. Epub 2011/08/06. doi: 10.1186/1471-2105-12-322. PubMed PMID: 21816037; PMCID: PMC3166942.

75. Song W-M, Di Matteo T, Aste T. Building complex networks with Platonic solids. Physical Review E. 2012;85(4):046115.

76. Song W-M, Di Matteo T, Aste T. Nested hierarchies in planar graphs. Discrete Applied Mathematics. 2011;159(17):2135-46.

77. Song WM, Zhang B. Multiscale Embedded Gene Co-expression Network Analysis. PLoS Comput Biol. 2015;11(11):e1004574. Epub 2015/12/01. doi: 10.1371/journal.pcbi.1004574. PubMed PMID: 26618778; PMCID: PMC4664553.

78. Zhang B, Horvath S. A general framework for weighted gene co-expression network analysis. Stat Appl Genet Mol Biol. 2005;4:Article17. Epub 2006/05/02. doi: 10.2202/1544-6115.1128. PubMed PMID: 16646834.

79. Tumminello M, Aste T, Di Matteo T, Mantegna RN. A tool for filtering information in complex systems. Proc Natl Acad Sci U S A. 2005;102(30):10421-6. Epub 2005/07/20. doi: 10.1073/pnas.0500298102. PubMed PMID: 16027373; PMCID: PMC1180754.

80. Tumminello M, Aste T, Di Matteo T, Mantegna RN. A tool for filtering information in complex systems. Proceedings of the National Academy of Sciences of the United States of America. 2005;102(30):10421-6.

81. Song WM, Di Matteo T, Aste T. Building complex networks with Platonic solids. Phys Rev E Stat Nonlin Soft Matter Phys. 2012;85(4 Pt 2):046115. Epub 2012/06/12. doi: 10.1103/PhysRevE.85.046115. PubMed PMID: 22680546.

82. Barabasi AL, Oltvai ZN. Network biology: understanding the cell's functional organization. Nat Rev Genet. 2004;5(2):101-13. Epub 2004/01/22. doi: 10.1038/nrg1272. PubMed PMID: 14735121.

83. Song WM, Lin X, Liao X, Hu D, Lin J, Sarpel U, Ye Y, Feferman Y, Labow DM, Walsh MJ, Zheng X, Zhang B. Multiscale network analysis reveals molecular mechanisms and key regulators of the tumor microenvironment in gastric cancer. Int J Cancer. 2019. Epub 2019/08/30. doi: 10.1002/ijc.32643. PubMed PMID: 31463974.

84. Seidman SB. Network structure and minimum degree. Social networks. 1983;5(3):269-87.

85. Zhang B, Gaiteri C, Bodea LG, Wang Z, McElwee J, Podtelezhnikov AA, Zhang C, Xie T, Tran L, Dobrin R, Fluder E, Clurman B, Melquist S, Narayanan M, Suver C, Shah H, Mahajan M, Gillis T, Mysore J, MacDonald ME, Lamb JR, Bennett DA, Molony C, Stone DJ, Gudnason V, Myers AJ, Schadt EE, Neumann H, Zhu J, Emilsson V. Integrated systems approach identifies genetic nodes and networks in late-onset Alzheimer's disease. Cell. 2013;153(3):707-20. Epub 2013/04/30. doi: 10.1016/j.cell.2013.03.030. PubMed PMID: 23622250; PMCID: PMC3677161.

86. Benjamini Y, Hochberg Y. Controlling the False Discovery Rate - a Practical and Powerful Approach to Multiple Testing. J R Stat Soc B. 1995;57(1):289-300. PubMed PMID: WOS:A1995QE45300017.

87. Naseem M, Barzi A, Brezden-Masley C, Puccini A, Berger MD, Tokunaga R, Battaglin F, Soni S, McSkane M, Zhang W, Lenz HJ. Outlooks on Epstein-Barr virus associated gastric cancer. Cancer Treat Rev. 2018;66:15-22. Epub 2018/04/10. doi: 10.1016/j.ctrv.2018.03.006. PubMed PMID: 29631196; PMCID: PMC5964025.

1. [↑](#endnote-ref-1)
